# Targeting miR-423-5p Reverses Exercise Training–Induced HCN4 Channel Remodeling and Sinus Bradycardia

**DOI:** 10.1161/CIRCRESAHA.117.311607

**Published:** 2017-10-12

**Authors:** Alicia D’Souza, Charles M. Pearman, Yanwen Wang, Shu Nakao, Sunil Jit R.J. Logantha, Charlotte Cox, Hayley Bennett, Yu Zhang, Anne Berit Johnsen, Nora Linscheid, Pi Camilla Poulsen, Jonathan Elliott, Jessica Coulson, Jamie McPhee, Abigail Robertson, Paula A. da Costa Martins, Ashraf Kitmitto, Ulrik Wisløff, Elizabeth J. Cartwright, Oliver Monfredi, Alicia Lundby, Halina Dobrzynski, Delvac Oceandy, Gwilym M. Morris, Mark R. Boyett

**Affiliations:** From the Division of Cardiovascular Sciences, University of Manchester, United Kingdom (A.D., C.M.P., Y.W., S.N., S.J.R.J.L., C.C., H.B., Y.Z., J.E., A.R., A.K., E.J.C., O.M., H.D., D.O., G.M.M., M.R.B.); K.G. Jebsen Center for Exercise in Medicine, Department of Circulation and Medical Imaging, Faculty of Medicine and Health Sciences, Norwegian University of Science and Technology, Trondheim, Norway (A.B.J., U.W.); Faculty of Health Sciences, NNF Center for Protein Research, University of Copenhagen, Denmark (N.L., P.C.P., A.L.); School of Healthcare Science, Manchester Metropolitan University, United Kingdom (J.C., J.M.); Department of Cardiology, CARIM School for Cardiovascular Diseases, Faculty of Health, Medicine and Life Sciences, Maastricht University, Netherlands (P.A.d.C.M.); and School of Human Movement & Nutrition Sciences, University of Queensland, Australia (U.W.).

**Keywords:** athletes, exercise training, ion channel remodeling, micro-RNAs, sinoatrial node, sinus bradycardia

## Abstract

**Rationale:**

Downregulation of the pacemaking ion channel, HCN4 (hyperpolarization-activated cyclic nucleotide gated channel 4), and the corresponding ionic current, *I*_f_, underlies exercise training–induced sinus bradycardia in rodents. If this occurs in humans, it could explain the increased incidence of bradyarrhythmias in veteran athletes, and it will be important to understand the underlying processes.

**Objective:**

To test the role of HCN4 in the training-induced bradycardia in human athletes and investigate the role of microRNAs (miRs) in the repression of HCN4.

**Methods and Results:**

As in rodents, the intrinsic heart rate was significantly lower in human athletes than in nonathletes, and in all subjects, the rate-lowering effect of the HCN selective blocker, ivabradine, was significantly correlated with the intrinsic heart rate, consistent with HCN repression in athletes. Next-generation sequencing and quantitative real-time reverse transcription polymerase chain reaction showed remodeling of miRs in the sinus node of swim-trained mice. Computational predictions highlighted a prominent role for miR-423-5p. Interaction between miR-423-5p and HCN4 was confirmed by a dose-dependent reduction in HCN4 3′-untranslated region luciferase reporter activity on cotransfection with precursor miR-423-5p (abolished by mutation of predicted recognition elements). Knockdown of miR-423-5p with anti-miR-423-5p reversed training-induced bradycardia via rescue of HCN4 and *I*_f_. Further experiments showed that in the sinus node of swim-trained mice, upregulation of miR-423-5p (intronic miR) and its host gene, NSRP1, is driven by an upregulation of the transcription factor Nkx2.5.

**Conclusions:**

HCN remodeling likely occurs in human athletes, as well as in rodent models. miR-423-5p contributes to training-induced bradycardia by targeting HCN4. This work presents the first evidence of miR control of HCN4 and heart rate. miR-423-5p could be a therapeutic target for pathological sinus node dysfunction in veteran athletes.

## Abstract 2

Supplemental Digital Content is available in the text.

Athletes are prone to cardiac arrhythmias, and sinus bradycardia is the most common rhythm disturbance.^1^ In the long term, this physiological adaptation can become pathological because veteran athletes are more likely to have sinus node dysfunction and to need an electronic pacemaker implantation than nonathletes.^2–4^ In 2 rodent models of exercise training, we previously demonstrated that the training-induced bradycardia is predominantly the result of a downregulation of the key pacemaking ion channel HCN4 (hyperpolarization-activated cyclic nucleotide gated channel 4) and the corresponding ionic current (funny current, *I*_f_) in the sinus node.^5^ We now report the first evidence that HCN4 and *I*_f_ downregulation is also responsible for training-induced sinus bradycardia in human athletes. What is responsible for the downregulation of HCN4 in the athlete? Despite its fundamental importance, regulation of HCN4 in health and disease is poorly understood. MicroRNAs (miRs) have been shown to play pivotal roles in cardiac remodeling in a variety of settings by post-transcriptional silencing of genes.^6–9^ Some miRs have been implicated in ion channel remodeling.^7–9^ We report here that the downregulation of HCN4 and *I*_f_ in trained mice is the result of an upregulation of miR-423-5p in the sinus node, and the training-induced bradycardia can be reversed by targeting the miR-dependent HCN4 remodeling.

**Editorial, see p 1027**

**Meet the First Author, see p 1022**

## Methods

A 3-lead ECG was recorded from male volunteers aged 18 to 30 years: 8 competitive endurance athletes and 10 sedentary age-matched (control) subjects. The heart rate was measured before and after complete autonomic blockade (achieved by intravenous injection of 0.04 mg/kg atropine and 0.2 mg/kg propranolol followed by top-up doses). After complete autonomic blockade, 7.5 mg ivabradine was administered orally, and the change in heart rate was recorded and used as a measure of the involvement of *I*_f_ in pacemaking. Ten-week-old C57BL/6J mice were trained by swimming for 60 minutes twice daily for 28 days.^5^ miR, mRNA, and protein expression in sinus node biopsies was measured by next-generation sequencing, quantitative real-time reverse transcription polymerase chain reaction (qPCR), Western blot, and high-resolution mass spectrometry. Computational predictions, luciferase reporter gene assays, and in vitro overexpression studies were used to identify miRs and transcription factors capable of regulating expression. The role of a candidate miR in the training-induced bradycardia was tested in vivo by administering an appropriate cholesterol-conjugated anti-miR.^6^ ECG recording, in vitro tissue electrophysiology, Western blot, sinus node cell isolation, and whole-cell patch clamp were used to characterize the mice and study HCN4 and *I*_f_ remodeling. Statistically significant differences were determined using an appropriate test; *P*<0.05 was regarded as significant. In figures, bar charts show means±SEM. Further details of methods are available in the Online Data Supplement.

## Results

### Evidence of *I*_f_ Remodeling in Human Athletes

**Figure 1.**
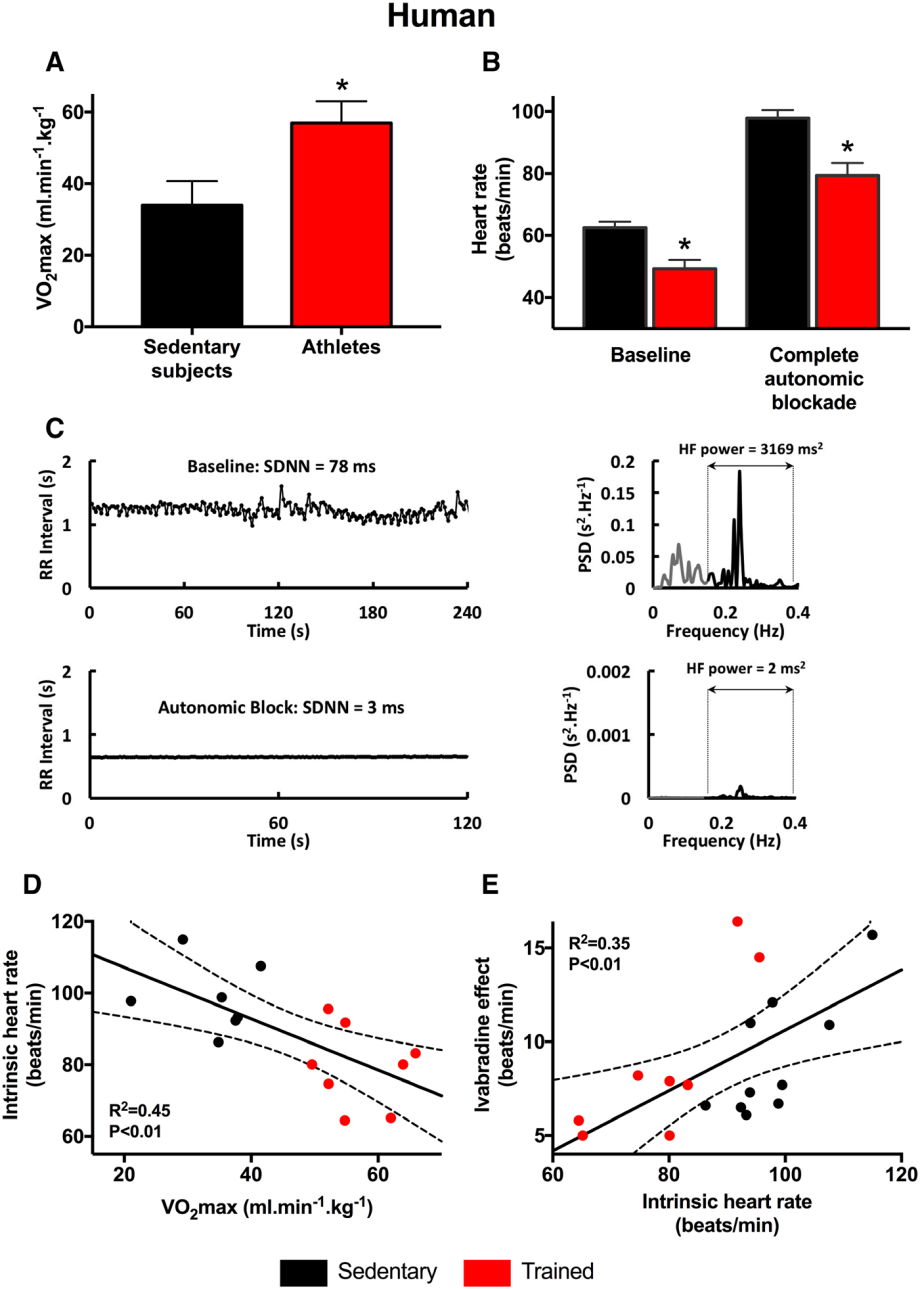
**Evidence of a role for HCN4 (hyperpolarization-activated cyclic nucleotide gated channel 4) in the resting bradycardia in human athletes.** **A**, VO_2_max of sedentary human subjects and human athletes (n=7/8). **B**, Heart rates measured under baseline conditions and after complete autonomic blockade of sedentary human subjects and human athletes (n=10/8). **C**, Complete autonomic blockade abolishes heart rate variability. **Left**, Examples traces of RR interval under baseline conditions and after complete autonomic blockade. **Right**, Spectral analyses of corresponding RR interval plots demonstrating near-abolition of high-frequency (HF) heart rate variability after autonomic blockade (note difference in *y* axis scale). **D**, Relationship between the intrinsic heart rate measured after complete autonomic blockade and VO_2_max in sedentary human subjects and human athletes (n=7/8). **E**, Relationship between ivabradine-induced decrease in heart rate and the intrinsic heart rate measured after complete autonomic blockade in sedentary human subjects and human athletes (n=10/8). In **D** and **E**, data fit by linear regression; best fit line, 95% confidence limits, and *R*^2^ and *P* values shown. PSD indicates power spectral density; and SDNN, standard deviation of normal to normal beats.

Experiments were conducted on groups of human male nonathletes (n=10) and athletes (n=8). The characteristics of the 2 groups are given in Online Table I. As expected, the maximum O_2_ uptake (VO_2_max, a measure of fitness) of the athletes was significantly higher than that of the nonathletes (Figure 1A). Figure 1B shows the resting heart rate under baseline conditions of the 2 groups of subjects; as expected, the heart rate of the athletes was significantly lower. It is still widely thought that the training-induced bradycardia is the result of an increase in vagal tone based on reported increases in heart rate variability in athletes^10^; heart rate variability is considered a measure of autonomic tone. However, we have recently shown that heart rate variability is primarily determined by heart rate and not autonomic tone.^11^ In the present study, neither uncorrected standard deviation of normal to normal beats (a measure of heart rate variability) or standard deviation of normal to normal beats corrected for changes in heart rate^11^ was increased in the athletes (Online Table I). Figure 1B also shows the intrinsic heart rate after complete autonomic blockade in the 2 groups of subjects. Autonomic blockade was achieved by intravenous injection of 0.2 mg/kg propranolol and 0.04 mg/kg atropine, doses that have previously been demonstrated to cause complete autonomic blockade in human subjects.^12,13^ The intrinsic heart rate after complete autonomic blockade in the nonathletes was 97.9±2.6 bpm, similar to the value reported by Jose and Collison^14^ of 105.6±0.6 bpm from 139 untrained male subjects 20 to 30 years of age. This suggests that complete autonomic blockade was achieved (see Online Data Supplement for further discussion).^15^ Furthermore, heart rate variability was almost completely eliminated after autonomic blockade: uncorrected standard deviation of normal to normal beats was reduced by 94%, corrected standard deviation of normal to normal beats was reduced by 90%, and high-frequency spectral heart rate variability was reduced by >99% (Figure 1C). This is consistent with complete autonomic blockade. Figure 1B shows that there was a significant bradycardia in the athletes (as compared with the nonathletes) after complete autonomic blockade, as well as under baseline conditions (the relative bradycardia was larger after complete autonomic blockade). We conclude that the bradycardia cannot be attributed to the autonomic nervous system, although we appreciate that others have concluded that it is the result of high vagal tone (see Online Data Supplement for further discussion). Figure 1D shows that the intrinsic heart rate of the nonathletes and athletes is significantly correlated with the fitness of the subjects as measured by the VO_2_max. Figure 1E shows a significant correlation between the heart rate lowering effect of oral ivabradine (blocks HCN4 and *I*_f_) and the intrinsic heart rate of the nonathletes and athletes: subjects with a lower intrinsic heart rate (generally athletes) had a blunted response to ivabradine. This suggests that in the human athlete, there is a downregulation of HCN4 and *I*_f_, and this could be the cause (or at least a key contributing cause) of the training-induced bradycardia.

### Training-Induced Downregulation of HCN4 and Dysregulation of miRs

**Figure 2.**
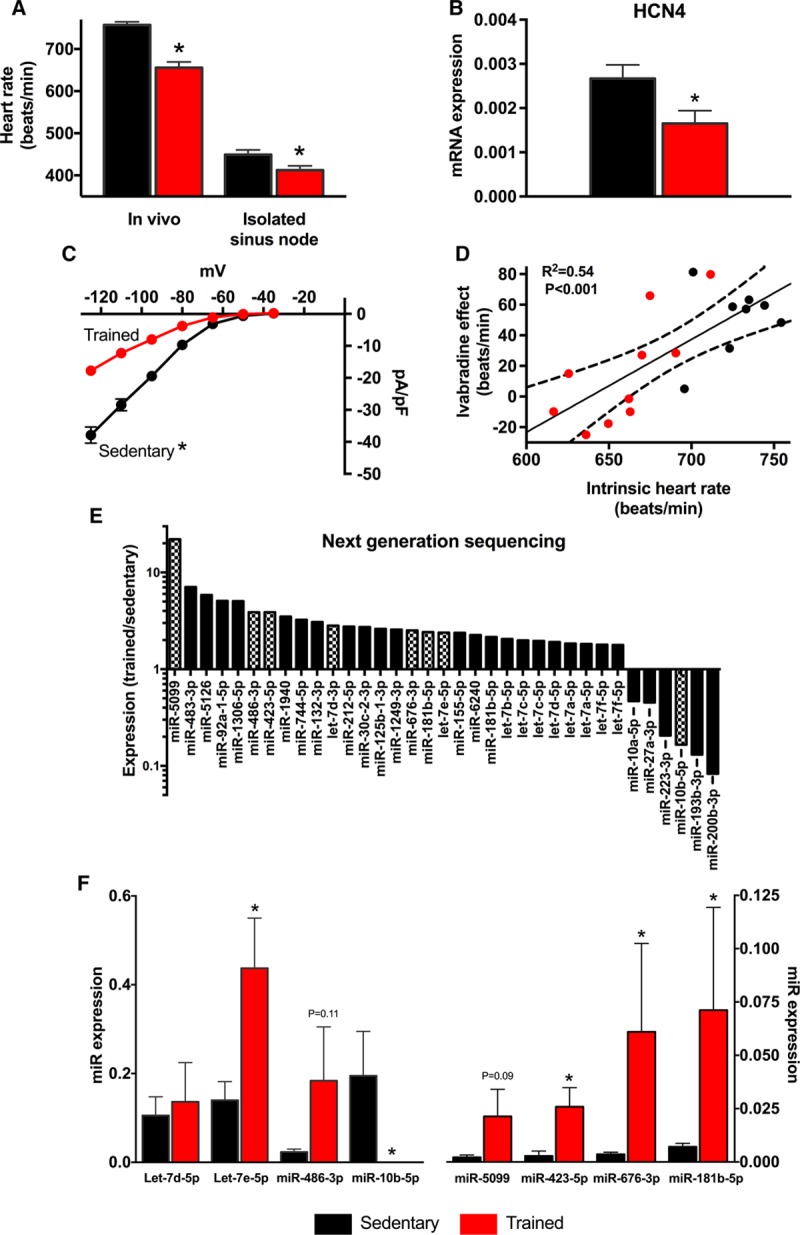
***I*_f_ remodeling in training-induced bradycardia is accompanied by dysregulation of miRs.** **A**, Heart rates of sedentary and trained mice measured in vivo in conscious animals (n=5/9) and in vitro in isolated sinus node preparations (n=6/6). **B**, Expression of HCN4 (hyperpolarization-activated cyclic nucleotide gated channel 4) mRNA in sinus node of sedentary and trained mice (n=5/5). **C**, Current–voltage relationships for *I*_f_ recorded from single sinus node cells from sedentary (n=47 cells/5 mice) and trained (n=58 cells/4 mice) mice. **D**, Relationship between ivabradine-induced decrease in heart rate and the intrinsic heart rate measured in vivo after complete autonomic blockade in sedentary and trained mice (n=8/10). Data fit by linear regression; best fit line, 95% confidence limits, and *R*^2^ and *P* values are shown. **E**, Significant (as determined by DeSeq) training-induced changes in miR expression (measured by next generation sequencing) in sinus node of mice. Ratio of miR expression in trained mice to expression in sedentary mice shown on logarithmic scale. **Hatched bars** indicate significant differences after Benjamini–Hochberg false discovery rate (FDR) correction (*P*<0.05). Data obtained from 3 pooled RNA samples per group (n=2/2). **F**, Verification of training-induced changes in miRs by quantitative real-time reverse transcription polymerase chain reaction (qPCR). Expression shown in sedentary and trained mice (n=6/7–9). *Significant difference between sedentary and trained data (*P*<0.05).

After swim-training for 4 weeks in the mouse, there was a significant bradycardia both in vivo and in the isolated sinus node (Figure 2A). This was confirmed by intracellular action potential recording from the isolated sinus node; the bradycardia was accompanied by a small positive shift of the maximum diastolic potential and prolongation of the action potential (Online Table II). The 2 main pacemaking mechanisms are the membrane and Ca^2+^ clocks.^16^ Although the main Ca^2+^ clock transcripts in the sinus node were unaffected by training (Online Figure I), the transcript for HCN4 (the main component of the membrane clock^16^) was significantly downregulated in the sinus node after training (Figure 2B). As expected, this was accompanied by a reduction of *I*_f_ over a wide potential range in isolated sinus node cells from trained mice (Figure 2C). Consistent with this, Figure 2D shows that in the mouse as in the human (Figure 1E), there is a significant correlation between the heart rate lowering effect of ivabradine and the intrinsic heart rate: mice with a lower intrinsic heart rate (generally trained) had a blunted response to ivabradine. These data are consistent with a downregulation of HCN4 and *I*_f_ being the cause of the training-induced bradycardia in mice, consistent with our previous study.^5^

In sinus node biopsies, we measured expression of all miRs using next-generation sequencing. Seven hundred and fifteen miRs were detected in the sinus node. DeSeq analysis showed that 25 miRs were significantly increased >1.8 fold and 6 significantly decreased (Figure 2E; Online Table III). After applying a 5% Benjamini–Hochberg false discovery rate correction, miR-5099, miR-486-3p, miR-423-5p, Let-7d-3p, miR-676-3p, miR-181b-5p, and Let-7e-5p were significantly upregulated and miR-10b-5p downregulated (Figure 2E, hatched bars). qPCR analysis confirmed the majority of these changes (including in miR-423-5p; Figure 2F).

### HCN4 Is Target Gene for miR-423-5p

**Figure 3.**
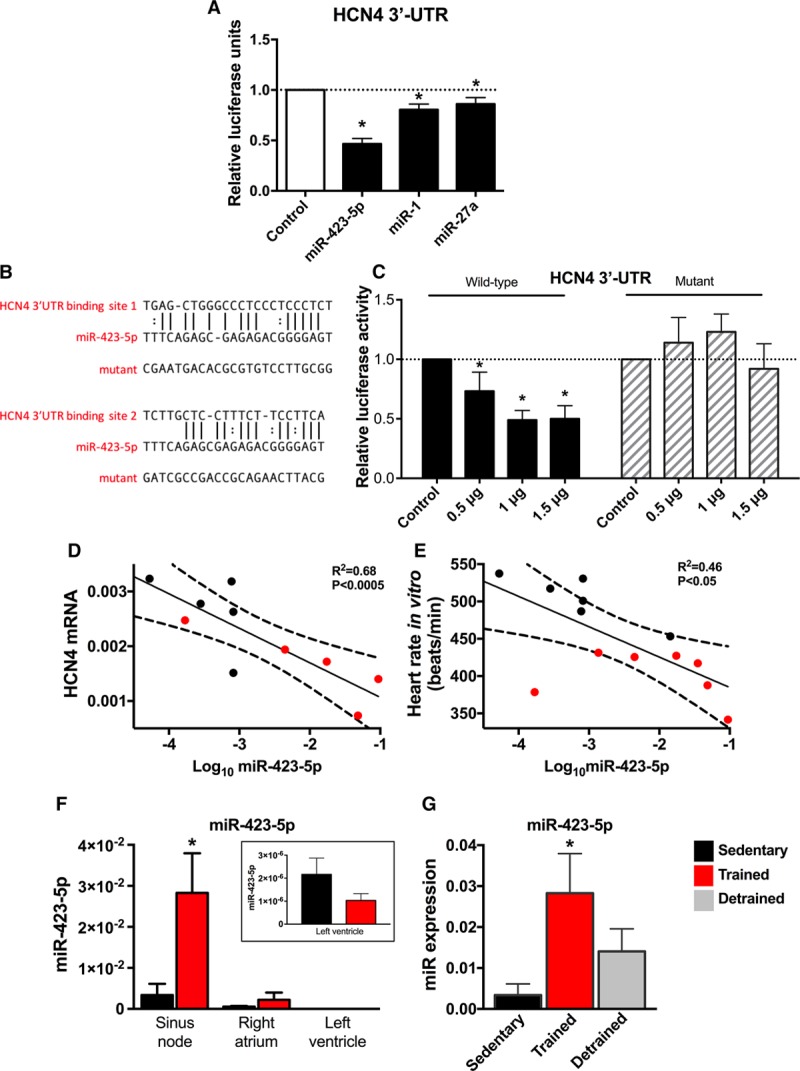
**HCN4 (hyperpolarization-activated cyclic nucleotide gated channel 4) is a target gene for miR-423-5p.** **A**, Luciferase reporter assay showing post-transcriptional repression of HCN4 by miRs. H9C2 cells were cotransfected with precursor miR and 3′-UTR of HCN4 cloned into expression vector downstream of luciferase gene. Luciferase activity is shown 24 h after cotransfection with different miRs, including a control (scrambled) miR. n=3 batches of cells with 4 to 5 replicates/batch. **B**, Predicted miR-423-5p binding sites in HCN4 3′-UTR and corresponding sequence of mutant HCN4 3′-UTR tested. **C**, Luciferase reporter assay showing dose-dependent repression of HCN4 by miR-423-5p and loss of repression by mutation of HCN4 3′-UTR. Luciferase activity is shown 24 h after cotransfection with different amounts of wild-type or mutant miR-423-5p. n=3 batches of cells with 4 replicates/batch. **D**, Relationship between HCN4 mRNA and log miR-423-5p in sedentary and trained mice (n=5/5). **E**, Relationship between the heart rate measured in vitro in the isolated sinus node and log miR-423-5p in sedentary and trained mice (n=6/7). In **D** and **E**, data fit by linear regression; best fit line, 95% confidence limits, and *R*^2^ and *P* values are shown. **F**, Expression of miR-423-5p (measured by quantitative real-time reverse transcription polymerase chain reaction [qPCR]) in atrium and ventricle is low and unaltered by training. Expression shown in sinus node, right atrial muscle, and left ventricular muscle from sedentary and trained mice (n=5/5/5). Inset, data from left ventricular muscle at magnified scale. **G**, Expression of miR-423-5p (measured by qPCR) in sinus node is partially restored on detraining (**right**). Expression shown in sinus node of sedentary, trained, and detrained mice (n=5/9/5). *Significantly different from control/sedentary data (*P*<0.05).

A computational search was conducted (see Online Data Supplement) to establish a link between significantly upregulated miRs and HCN4 downregulation. miRs bind cognate mRNAs by imprecise complementary base pairing to specific sequence motifs primarily in the 3′-untranslated region (UTR).^17^ Using the widely used algorithms RNA22,^18^ PITA,^19^ and TargetScan Mouse v7.1,^20^ we identified putative recognition sites for miR-423-5p (Online Figure II) and miR-486-3p (data not shown) within the mouse HCN4 3′-UTR sequence. To verify these predicted binding sites and experimentally establish HCN4 as a genuine target, we fused the HCN4 3′-UTR to a luciferase reporter gene (pHCN4-3′ UTR) and determined luciferase activity in H9c2 cells cotransfected with pHCN4-3′ UTR and synthetic precursors to miR-423-5p and miR-486-3p. Additionally, we included miR-1 and miR-27a in this analysis as we have previously found miR-1 to be upregulated in the sinus node of the trained mouse and rat,^5^ and all computational tools used indicated the presence of a highly conserved binding site for miR-27a. All miRs tested significantly supressed luciferase activity relative to a control (scrambled) miR, although suppression was modest on transfection with miR-1 and miR-486-3p (34%; data not shown) compared with miR-423-5p (Figure 3A). Surprisingly, miR-27a only produced a small suppression of luciferase activity (Figure 3A).

We selected miR-423-5p for further study because it was predicted to target HCN4, it was significantly upregulated using both next-generation sequencing (2.8-fold increase above baseline) and qPCR (8.1-fold increase above baseline), and it produced the largest suppression of luciferase activity among the miRs tested. We observed a dose-dependent effect of miR-423-5p on luciferase activity of pHCN4-3′ UTR (Figure 3C). Furthermore, mutation of predicted miR-423-5p binding sequences in the HCN4 3′- UTR (Figure 3B) abolished the effect of miR-423-5p on reporter expression (Figure 3C). These findings demonstrate a specific interaction between miR-423-5p and HCN4, and the predicted recognition elements identified in the HCN4 3′ -UTR contribute to this. Both HCN4 mRNA and intrinsic heart rate showed a significant inverse correlation with expression of miR-423-5p (Figure 3D and 3E). Linear regression analysis showed that the sinus node expression level of miR-423-5p explained 68% of the variation in HCN4 (Figure 3D; *R*^2^ = 0.68) and 46% of the variation in spontaneous beating rate of the sinus node (Figure 3E; *R*^2^ = 0.46). Upregulation of miR-423-5p appeared to be restricted to the trained sinus node—basal expression of miR-423-5p was lower in the atrium and ventricle and was unaltered by training (Figure 3F). After 2 weeks of detraining (after 4 weeks of training), there was a partial restoration of miR-423-5p (Figure 3G).

Although Figure 3A through 3C shows that miR-423-5p targets HCN4, it is possible that it has other targets. Previously, miR-423-5p has been reported to cause apoptosis of cardiomyocytes,^21–23^ and we performed the TUNEL (terminal deoxynucleotidyl transferase dUTP nick-end labeling) assay to assess apoptosis. However, we observed few apoptotic cells in the sinus node, and there was no significant difference in the number in sedentary and trained mice (Online Figure III).

### Training-Induced Downregulation of HCN4 and *I*_f_ and Resulting Sinus Bradycardia Is result of Upregulation of miR-423-5p

**Figure 4.**
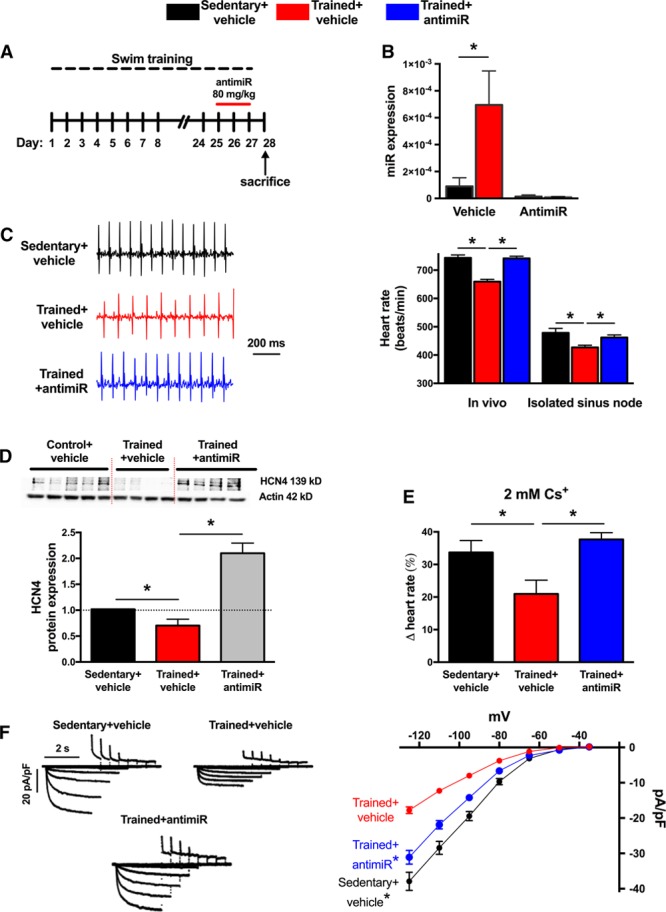
**Anti-miR to miR-423-5p reverses training-induced bradycardia and blunts HCN4 (hyperpolarization-activated cyclic nucleotide gated channel 4) channel remodeling.** **A**, Time course of exercise training and anti-miR administration. **B**, Anti-miR abolishes training-induced upregulation of miR-423-5p in sinus node. miR-423-5p (determined by quantitative real-time reverse transcription polymerase chain reaction [qPCR]) in sinus node of vehicle- or anti–miR-treated sedentary and trained mice shown (n=6/5/5/5). **C**, Anti-miR reverses training-induced bradycardia. Representative ECG traces (recorded from conscious animals) from a vehicle-treated sedentary mouse, vehicle-treated trained mouse, and anti–miR-treated trained mouse shown on **left** and mean heart rates (measured in vivo and in isolated sinus node) on **right** (n=10/12/12 and 9/12/12). **D**, Anti-miR reverses training-induced downregulation of HCN4. Western blots using antibodies recognizing HCN4 and actin (housekeeper) proteins for sinus node from vehicle-treated sedentary, vehicle-treated trained, and anti-miR-treated trained mice shown as well as mean expression level of HCN4 protein (normalized to actin) in the 3 groups (n=5/4/5 with 3 independent replicates per mouse). **E**, Anti-miR reverses training-induced downregulation in contribution of *I*_f_ to pacemaking. Percentage decrease in heart rate (recorded from isolated sinus node preparations) on blocking *I*_f_ using 2 mmol/L Cs^+^ invehicle-treated sedentary, vehicle-treated trained, and anti-miR-treated trained mice shown (n=6/7/4). **F**, Anti-miR reverses training-induced downregulation in *I*_f_. Representative *I*_f_ traces (normalized to cell capacitance) from vehicle-treated sedentary, vehicle-treated trained, and anti-miR-treated trained mice shown on **left** and mean current–voltage relationships for *I*_f_ from vehicle-treated sedentary (n=47 cells/5 animals), vehicle-treated trained (n=58 cells/4 animals), and anti-miR-treated trained (n=89 cells/5 animals) mice shown on **right**. *Significantly different (**B**–**E**) or significantly different from trained+vehicle data (**E**; *P*<0.05).

We hypothesized that the training-induced upregulation of miR-423-5p results in the downregulation of HCN4 and, consequently, a lower heart rate. To test this, miR-423-5p was knocked down in vivo via 3 daily intraperitoneal injections of cholesterol-conjugated anti-miR-423-5p (anti-miR) in sedentary mice and in trained mice at day 25 to 27 of the swimming protocol (Figure 4A). Administered in this way, cholesterol-conjugated anti-miR has previously been documented to be highly effective in knocking down target miR in the heart with long-lasting efficacy under in vivo conditions.^6^ At day 28, that is, 3 days after the first administration, qPCR analysis showed a dramatic reduction in the level of miR-423-5p (Figure 4B). A separate study demonstrated that the suppressive effect of the anti-miR on miR-423-5p persisted for up to 3 weeks after administration (Online Figure IV). Remarkably, the anti-miR abolished or blunted the training-induced bradycardia: it restored the heart rate measured in vivo and in vitro (isolated sinus node) to or toward the pretraining level (Figure 4C). Intriguingly, the anti-miR did not alter the heart rate in vivo in sedentary animals, despite successful knockdown of miR-423-5p (Online Figure V); the effect of the anti-miR was, therefore, restricted to the trained mouse. Western blot analysis demonstrated that the anti-miR completely restored total HCN4 protein in the sinus node (level in anti-miR-treated mice was 2.1× greater than that in vehicle-treated sedentary mice; Figure 4D). The effect of anti-miR on *I*_f_ was assessed in vitro by pharmacological block of *I*_f_ with 2 mmol/L Cs^+^ in the isolated sinus node (Figure 4E).^24^ Two mmol/L Cs^+^ produced a smaller decrease in spontaneous beating rate in trained animals (indicative of reduction in *I*_f_), and this effect was reversed by the anti-miR, indicating restoration of *I*_f_ by the anti-miR (Figure 4E). Whole cell patch clamp recordings from isolated sinus node cells confirmed this: the density of *I*_f_ was reduced in trained mice, and it was almost fully restored in trained mice treated with the anti-miR (Figure 4F). In summary, the anti-miR restored total HCN4 protein (transmembrane fraction of which is responsible for *I*_f_) beyond the control level, almost fully restored *I*_f_, fully restored responsiveness to Cs^+^ (indirect measure of *I*_f_), almost fully restored the intrinsic heart rate (arguably set by *I*_f_) measured in the isolated sinus node, and fully restored the heart rate measured in vivo (again arguably set by *I*_f_). Total HCN4 protein may have been restored beyond the control level because of an increase in the amount of protein being trafficked to the membrane. It is not known why heart rate in vivo was fully restored, whereas *I*_f_ was only partially restored—it is possible that there is an additional mechanism in operation. A control (nontargeting) anti-miR did not restore *I*_f_ (Online Figure VI). The heart weight:body weight ratio and various ECG parameters were largely unaffected by the anti-miR (Online Figure VII).

### Control of miR-423-5p in the Trained Sinus Node

**Figure 5.**
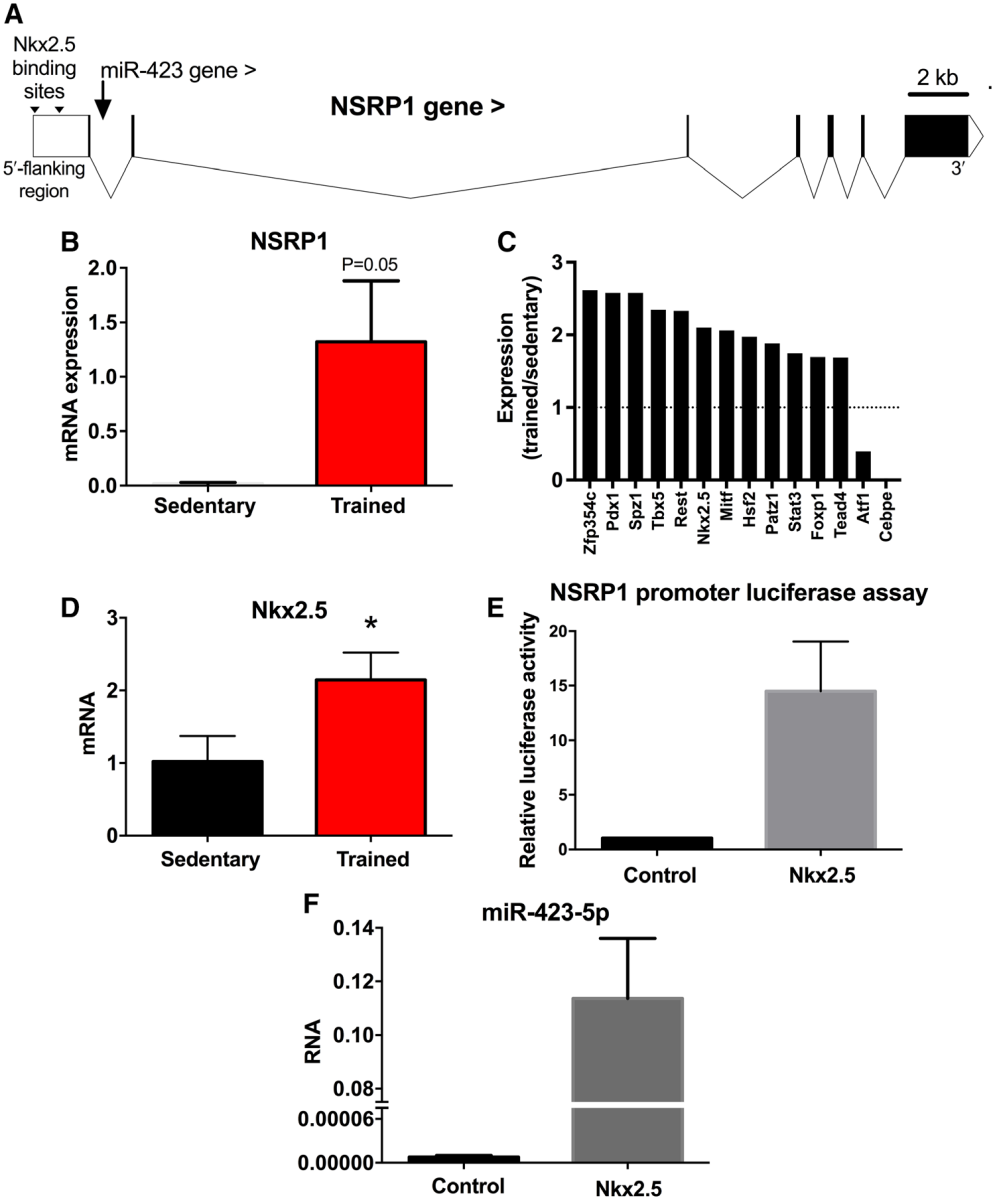
**Nkx2.5 regulation of miR-423-5p.** **A**, map of NSRP1 gene (solid blocks show exons with introns in between) showing location of Nkx2.5 binding sites and intronic location of the miR-423 gene. **B**, Expression of NSRP1 mRNA in sinus node of sedentary and trained mice (n=8/8). **C**, Significant (*P*<0.05) training-induced changes in expression of transcription factor transcripts (measured by quantitative real-time reverse transcription polymerase chain reaction [qPCR]) in sinus node of mice. Ratio of mRNA expression in trained mice to expression in sedentary mice shown (n=6/8). **D**, Expression of Nkx2.5 mRNA in sinus node of sedentary and trained mice (n=8/8). **E**, Luciferase reporter assay showing activation of NSRP1 transcription by Nkx2.5. H9c2 cells were transfected with 2.1 kb of the 5′ flanking region of NSRP1 cloned into expression vector downstream of gene for luciferase. The cells were cotransfected with Nkx2.5; control cells were not cotransfected with Nkx2.5. Luciferase activity is shown 48 h after transfection. n=3 independent batches of cells with 4 replicates/batch. **F**, Upregulation of miR-423-5p by Nkx2.5. miR-423-5p expression is shown in H9c2 cells not transfected (control) or transfected with Nkx2.5.

miR-423 is located in the first intron of its host gene, NSRP1, and both are transcribed in the same sense direction (Figure 5A). Therefore, it is possible that they are coregulated and share regulatory (promoter) elements, as has been shown previously for intronic miRs.^25,26^ qPCR confirmed that NSRP1 mRNA was significantly upregulated in the sinus node of trained mice (Figure 5B). A bioinformatics search using MatInspector revealed transcription factors that could potentially bind to the promoter region of NSRP1. The expression of the 88 top predicted transcription factors in the sinus node was investigated using qPCR—15 were significantly upregulated and 2 significantly downregulated after training (Figure 5C). Of these 17, Nkx2.5 (data for Nkx2.5 shown in more detail in Figure 5D) was the most promising on the basis that it was upregulated (therefore, potentially explaining the upregulation of NSRP1), it is an important cardiac transcription factor, it is known to work in consort with other transcription factors (Foxp1, Stat3, and Tbx5^27–29^) that were also upregulated in the sinus node with athletic training (Figure 5C), it represses the working phenotype in the sinus node, and most importantly it has previously been shown that upregulation is associated with sick sinus disease and sinus bradycardia.^30^ Although Nkx2.5 is thought not to be expressed in the adult sinus node, we observed substantial expression of both Nkx2.5 mRNA and protein in the sinus node even in the case of the sedentary mouse (Online Figure X; see Online Data Supplement for further discussion). Predicted Nkx2.5 binding sites on NSRP1 are given in Online Figure VIII. To verify NSRP1 as a target of Nkx2.5, we fused 2.1 kb of the 5′ flanking region of NSRP1 (upstream of the transcriptional start site) to luciferase and determined luciferase activity in H9c2 cells cotransfected with an Nkx2.5 overexpression plasmid. Nkx2.5 significantly increased luciferase activity relative to vehicle control (Figure 5E). To confirm this finding, Nkx2.5 was then overexpressed in H9c2 cells. Surprisingly, Nkx2.5 had little effect on expression of NSRP1 (Online Figure IX), but it resulted in a robust increase in miR-423-5p (Figure 5F). It is possible that miR-423-5p is one of 20% of intronic miRs that are predicted to inhibit expression of their host gene.^31^ In this case, whether Nkx2.5 upregulates both NSRP1 and miR-423-5p may depend on the precise circumstances. We conclude from these data that the upregulation of miR-423-5p in the sinus node after training involves an upregulation of the transcription factor, Nkx2.5.

## Discussion

Our previous work on rodents was the first to attribute exercise training–induced bradycardia to the downregulation of HCN4 and *I*_f_.^5^ We now present evidence suggesting this is also the case in human athletes. In addition, we show that HCN4 is a novel target for post-transcriptional repression by miR-423-5p and the training-induced downregulation of HCN4 and *I*_f_, and consequently, the bradycardia is the result of an upregulation of Nkx2.5 and consequently miR-423-5p in the sinus node. This is the first report of miR-dependent regulation of pacemaking and heart rate.

There is widespread belief that the resting bradycardia in athletes is the result of high vagal tone, although vagal tone has never been directly measured in athletes.^32^ However, critical analysis of the literature does not necessarily support this.^15,33,34^ An increase in heart rate variability in human athletes has been extensively quoted as evidence for high vagal tone in athletes.^32^ However, we have shown from an analysis of the underlying biophysics of pacemaking that heart rate variability is primarily determined (in an exponential-like manner) by heart rate, and the increase in heart rate variability in athletes is attributed to the resting bradycardia rather than any increase in vagal tone.^11^ This study has shown that the relative bradycardia in human athletes (as compared with the heart rate of the control subjects) was still present (and in fact was larger) after complete autonomic blockade (Figure 1B). The same result (a larger relative bradycardia after autonomic blockade) has been reported by 3 other studies of human athletes.^15^ This suggests that the autonomic nervous system is not responsible for the resting bradycardia. Once again, many studies of animal models of exercise training show no evidence of high vagal tone.^15^ However, some studies of human athletes or animal models of exercise training apparently show a reduction of the relative bradycardia after autonomic blockade, leaving open the possibility that high vagal tone does play a role.^15,35,36^ This is considered further in Discussion in the Online Data Supplement. Previously, we showed that the reduction of heart rate on blocking *I*_f_ by ivabradine or Cs^+^ is decreased in the trained rodent, and this is attributed to the training-induced downregulation of HCN4 and *I*_f_.^5^ The corollary of this, as shown in Figure 2D, is that there is a correlation between the effect of ivabradine and the intrinsic heart rate of sedentary and trained mice. As shown in Figure 1E, the same correlation is observed in sedentary and trained human subjects. It is, therefore, possible that there is a training-induced downregulation of HCN4 and *I*_f_ in the human athlete, and this is the cause (or at least a contributing cause) of the bradycardia.

miR profiling by next-generation sequencing revealed 35 miRs to be altered by endurance training in the sinus node (Figure 2E). Several of these miRs have previously been shown to exert regulatory effects in the heart: miR-486-3p, upregulated in the sinus node of the trained mice (Figure 2E and 2F), has previously been shown to be upregulated in the hearts of swim-trained mice and involved in the antifibrotic effects of exercise^37^; Let-7e, upregulated in the sinus node of the trained mice (Figure 2E and 2F), has previously been shown to have an antiarrhythmic effect mediated via a downregulation of the β1 adrenergic receptor in myocardial infarction rats^38^; finally, miR-10b-5p, downregulated in the sinus node of the trained mice (Figure 2E and 2F), has previously been shown to regulate the key cardiac transcription factor Tbx5, known to be involved with the cardiac conduction system.^39^ Previously, the plasma level of miR-423-5p has been reported to be elevated in heart failure, acute myocardial infarction, stable coronary artery disease, and patients undergoing cardiac surgery.^40–42^ In the case of heart failure at least, this is thought to be the result of altered myocardial expression.^42^ In these studies, although the source of miR-423-5p may be the myocardium, it is not known from what part of the heart it originates. Figure 3F suggests that miR-423-5p is preferentially expressed by the sinus node, and this raises the question of whether the sinus node is the source of miR-423-5p in heart failure. Paradoxically, the sinus node in heart failure is like that in the athlete; there is intrinsic sinus bradycardia and a widespread remodeling of the sinus node with a downregulation of ion channels.^43–45^ miRs are known to function according to a combinatorial circuitry model, whereby a single miR targets multiple mRNAs and several coexpressed miRs may target a single mRNA.^46^ While our data suggest a prominent role for miR-423-5p in regulating HCN4, we cannot rule out a role for other miRs. Ultimately, regulation of heart rate is a complex and dynamic process involving acute regulation by the autonomic nervous system and longer-term regulation involving changes in ion channel expression brought about by transcription factors and, as shown for the first time by our study, miRs.

In conclusion, our findings provide new insight into the molecular mechanisms underlying the sinus bradycardia in athletes, and this may have implications for other conditions in which *I*_f_ is dysregulated, for example, heart failure.^43^ Although the changes in the sinus node and sinus bradycardia are well tolerated by healthy young athletes, this is not necessarily the case in some veteran athletes and the changes manifest as pathological sinus node dysfunction—the incidence of pacemaker implantation in veteran athletes is higher than that in nonathletes.^2–4^ Inhibition of miR-423-5p (especially because upregulation of miR-423-5p could be restricted to the sinus node; Figure 3F) could be an alternative therapeutic strategy to electronic pacemaker implantation for pathological sinus node dysfunction seen in some veteran athletes.

## Sources of Funding

This work was supported by the British Heart Foundation (PG/14/24/30626, RG/11/18/29257, and PG/13/99/30233), a personal fellowship to A. D’Souza from the International Society for Heart Research and Servier, and support from the Danish Council for Independent Research (DFF-4092-00045) to A. Lundby.

## Disclosures

None.

## Supplementary Material

## Novelty and Significance

### What Is Known?

- Athletes have a slow resting heart rate, that is, sinus bradycardia, and in veteran athletes, this can necessitate electronic pacemaker implantation.
- We have previously shown that a downregulation of the key pacemaking ion channel, HCN4 (hyperpolarization-activated cyclic nucleotide gated channel 4), and the corresponding ionic current (funny current or *I*_f_) in the sinus node underlies the sinus bradycardia in rodent models of exercise training.
