# Supplementary material for: Targeting miR-423-5p Reverses Exercise Training–Induced HCN4 Channel Remodeling and Sinus Bradycardia
Source: Circ Res. 2017 Oct 12;121(9):1058–68. doi: 10.1161/CIRCRESAHA.117.311607 (PMC5636198; doi:10.1161/CIRCRESAHA.117.311607)
Supplement: Supplementary file 1 [file res-121-1058-s001.pdf]

Supplemental Material

Targeting miR-423-5p reverses exercise training-induced HCN4 channel remodelling and sinus bradycardia

Alicia D'Souza, Charles Pearman, Yanwen Wang, Shu Nakao, Sunil Jit R.J. Logantha, Charlotte Cox, Hayley Bennett, Yu Zhang, Anne Berit Johnsen, Nora Linscheid, Pi Camilla Poulsen, Jonathan Elliot, Jessica Coulson, Jamie McPhee, Abigail Robertson, Paula Da Costa Martins, Ashraf Kitmitto, Ulrik Wisloff, Elizabeth J. Cartwright, Oliver Monfredi, Alicia Lundby, Halina Dobrzynski, Delvac Oceandy, Gwilym M. Morris, Mark R. Boyett

SUPPLEMENTAL METHODS

Human study

Study subjects were male volunteers aged between 18 and 30 years and non-smokers with no known illnesses and taking no medications. The study group were competitive endurance athletes of a good standard, training or competing for a minimum of eight h per week. Control subjects were sedentary age-matched males exercising for less than 2 h per week. Table S1 shows further characteristics of the subjects. The study protocol was approved by the regional research ethics committee and subjects gave informed consent. A three lead ECG was continuously recorded using an AD Instruments Powerlab ECG system. Subjects were studied in a supine position and in the post-absorptive fasted state. Complete autonomic blockade for measurement of the intrinsic heart rate was achieved by intravenous injection of atropine 0.04 mg/kg and propranolol 0.2 mg/kg. Ivabradine 7.5 mg p.o. was given to block the funny current, I_f. At peak plasma ivabradine (1 h), further atropine and propranolol were administered to ensure continued complete autonomic block. The additional dose calculations were based on known pharmacokinetics of atropine and propranolol:

$$C_2 = C_1 e^{-(0.693/t_{1/2})t}$$

where C_2 is the plasma concentration, C_1 is the original plasma concentration, $t^{1/2}$ is the half time of the drugs (4 h) and t is elapsed time since the original injection (1 h). The effect of block of I_f on the intrinsic heart rate was measured at this time. Heart rate was taken as an average over two 5-min periods (baseline) and over a single 5-min period (intrinsic heart rate; intrinsic heart rate+ivabradine). Heart rate variability was assessed in the following manner: continuous single lead ECGs were recorded digitally at 10 kHz using LabChart v7.0. 256 s ECG segments were selected after an appropriate acclimatisation period following each intervention (10 minutes pre-resting ECG, 5 min following intravenous atropine and propranolol, 60 min following oral ivabradine). R wave peaks were identified automatically and RR intervals were exported to Kubios v2.0 for analysis of heart rate variability. RR series were interpolated at 4 Hz. Heart rate variability was assessed in the time and frequency domains with the high frequency band defined as 0.15-0.4 Hz.

Experimental animals

Care and use of laboratory animals conformed to the UK Animals (Scientific Procedures) Act 1986. Ethical approval for all experimental procedures was granted by the University of Manchester. Eight-week-old male C57BL/6J mice (Harlan Laboratories; initial body weight, 20–25 g) were randomly assigned to either sedentary or trained groups. Mice were housed five per cage in a temperature-controlled room (22°C) with a 12 h:12 h light:dark lighting regime and free access to food and water.

Swim training

Mice were subjected to a swimming programme described previously.[1, 2] The mice were swim-trained for 60 min twice daily for 28 consecutive days. All mice were able to complete the course of training. Age- and weight-matched sedentary littermates served as controls for all experimental conditions and were handled daily. Additionally, a cohort of swim-trained mice was submitted to detraining for two weeks after the 28-day training period during which physical activity was restricted to the space of the cage.

Conscious ECG recordings

ECGs were recorded non-invasively in unrestrained, conscious mice using the ECGenie recording enclosure (Mouse Specifics, Inc., Boston, MA, USA) as described previously.[1] Heart rate was measured over 100 consecutive beats. The effect of (6 mg kg^{-1}) ivabradine under complete autonomic block with atropine (0.5 mg kg^{-1}) and propranolol (1 mg kg^{-1}) was measured as previously described.[1]

Unconscious ECG recordings

ECGs were recorded under isofluorane anaesthesia as described previously.[1] 1.5% isoflurane in 100% O_2 with a flow rate of 1 l/min was used. ECG parameters were measured over 100 consecutive beats.

AntimiR design and administration

We employed a previously validated chemistry and administration protocol that has been shown to be highly efficient in knocking down target micro-RNAs (miRs) in the heart with long-lasting efficacy under *in vivo* conditions.[3] Chemically modified antisense nucleotide (antimiR) against miR-423-5p was designed and obtained from Integrated DNA Technology (IDT, Belgium). The sequence of antimiR-423-5p was the exact antisense of the mature miR sequence (obtained from miRbase[4]): 5′-UGAGGGGCAGAGAGCGAGACUUU/3CholTEG with 3′ cholesterol conjugation, two phosphorothioate bonds at the very first 5′ end and four phosphorothioate bonds between the last 3′ bases. These modifications improve cell uptake and protect against nuclease degradation while increasing affinity of the antisense oligonucleotide to the target.[5] Chemically modified antisense oligonucleotides designed to target *C. elegans* miR-39-5p (5′-AAGGCAAGCUGACCCUGAAGUU-3′/3CholTEG-3′) that does not target mammalian sequences[3] was used as a control antimiR. AntimiRs were diluted in sterile saline and administered to mice via intraperitoneal injection on three consecutive days (each day, 80 mg/kg). Vehicle-treated mice were given an equivalent volume of sterile saline.

RNA isolation

Mice were killed by cervical dislocation and a ~1 mm biopsy collected from the sinus node at the level of the main branch from the crista terminalis and a separate biopsy was collected from the neighbouring right atrial free wall. Biopsies were frozen in liquid N_2 and stored at −80°C until use. Total RNA was isolated using an RNeasy Micro kit (Qiagen) according to the manufacturer's instructions. Total RNA was isolated from H9c2 cells using Trizol Reagent in conjunction with Purelink RNA Mini Kit (Life Technologies) according to the manufacturer's instructions. RNA purity and quantity was determined using a NanoDrop ND-1000 spectrophotometer (NanoDrop Technologies, Wilmington, DE, USA).

Quantitative PCR (qPCR) for miRs

miR expression levels were measured using miRCURY LNA (Locked Nucleic Acid) Universal RT microRNA PCR setup (Exiqon, Denmark) using the manufacturer's instructions for cDNA synthesis and qPCR. Primers were purchased from Exiqon (miR-10b-5p, 205637; miR-486-3p, 204107; miR-423-5p, 205624; miR-676-3p, 205098; miR-181b-5p, 204530; Let-7e-5p, 205711; Let-7d-5p, 204124). Primer set for mmu-miR-5099 was custom designed according to previously published sequences. Expression of miR was calculated by the ΔCt method and normalisation to expression of RNU1A1, which was determined as the optimal endogenous control (RNU1A1, SNORD65 and RNU5G were tested) using the algorithm geNorm (qBase^{plus}, version 2.0, Biogazelle, Belgium).

qPCR for mRNAs

First strand cDNA was synthesised using Superscript II reverse transcriptase (Invitrogen, Carlsbad, CA, USA). qPCR was performed using an ABI Prism 7900 HT Sequence Detection System (Applied Biosystems/Life Technologies Corporation, Carlsbad, CA, USA). The reaction mixture comprised 1 µl of cDNA, 1× Qiagen assay (HCN4, QT00268660; 18S, QT02448075), 1× SYBR Green Master Mix (Applied Biosystems) and DNase-free water. All samples were run in duplicate. The reaction conditions were: denaturation step of 95°C for 10 min followed by 40 cycles of amplification and quantification steps of 95°C for 30 s, 60°C for 30 s and 72°C for 1 min. The melt curve conditions were: 95°C for 15 s, 60°C for 15 s and 95°C for 15 s. mRNA expression was calculated by the ΔCt method and normalisation to the expression of 18S.

qPCR for transcription factors

Expression levels of seven housekeeping genes and 88 selected transcription factors predicted to target 2 kb of the NRSP1 5′ flanking region were measured using custom-designed TaqMan Low density array (TLDA) cards (Life Technologies, cat. no. 4342259; format 96A; transcripts studied are listed in Table S4) according to manufacturer's instructions. 200 ng of total RNA was used as a template in 20 µl reactions to generate cDNA using a High Capacity cDNA Reverse Transcription Kit (Life Technologies). cDNA was then combined with Universal Master Mix (Life Technologies) and applied to each port of a TLDA card organised in eight ports of 48 genes each. Thermal cycling was carried out on an ABI Prism 7900HT according to the manufacturer's recommended

protocol. Amplification plots were analysed using RQ manager (Life Technologies). Ct values were exported to RealTime Statminer (Integromics) data analysis package that enabled advanced filtering of outlier genes, geNorm-based selection of optimal endogenous controls genes Gapdh and Tbp and differential expression testing using the non-parametric Limma test.[6] Transcript expression levels were calculated using the ΔCt method.

Next generation sequencing

Four cDNA libraries (two for sedentary and two for trained groups) were constructed from three pooled samples each. The cDNA libraries were prepared from 1 μg of total RNA using TruSeq Small RNA Sample Prep Kit (Illumina, Inc.) according to the manufacturer's instructions. Briefly, RNA 3′ adapter and RNA 5′ RNA adapter were ligated to each end of small RNA molecules. The ligation products were used as a template for cDNA synthesis using SuperScript II Reverse Transcriptase (Invitrogen) to create single stranded cDNA. The cDNA was then PCR amplified using a common primer and a primer containing index sequences. After RT-PCR amplification, the cDNA libraries were purified by polyacrylamide gel electrophoresis to select libraries containing mature miR and other regulatory small RNAs. 22 and 30 nt bands were extracted from the gel and purified using the MinElute Gel Extraction Kit (Qiagen). The sizes of the selected library were validated by an Agilent Technologies 2100 Bioanalyzer using a High Sensitivity DNA chip.

50 base pair single-end reads were sequenced on the Illumina MiSeq sequencer (Illumina, Inc.) yielding up to 30 million raw reads per sample. Fastq files generated by MiSeq platform were analysed with FastQC (S. Andrews, 2010; *FastQC: a quality control tool for high throughput sequence data*; available online at: http://www.bioinformatics.babraham.ac.uk/projects/fastqc) and any low quality reads and contaminated barcodes and primers trimmed with Trimmomactic.[7] Reads without adaptor sequences and with no ambiguous bases and the final trimmed length of at least 19 nucleotides were included for the final alignment analysis. Libraries were then aligned to mm10 assembly of mouse genome using Tophat 2[8] which incorporates Bowtie, short-read aligner software. Alignments with the best score were reported from each read. The mapped reads were then counted against gff files downloaded from miRbase, mmu.gff3, with HTSeq.[9] Reads were considered as mature miRs if they fulfilled the 'Strict' requirement on HTSeq, i.e. mapped to and within the whole miR range as defined by miRbase. Normalisation (to control for the variation in the number of read sequences across samples) was done using the DESeq Bioconductor package in 'R' based on the geometric mean. After normalised read counts were obtained, differentially expressed miRs were identified by comparing sedentary versus trained samples with DESeq.[10] DESeq is based on the negative binomial distribution and outputs fold change and P values for differential expression. P values were then adjusted for multiple testing using a false discovery rate of 5% via the Benjamini-Hochberg method. miRs with $P<0.05$ were considered to be differentially expressed.

Computational prediction of miR targets and cis-acting transcription factors

We used three established miR target prediction algorithms to investigate whether any differentially expressed miRs identified by next generation sequencing (FDR<0.05) were predicted targets in the regulation of mouse HCN4 based on 3′ -UTR binding sites. miRs predicted by two out of three algorithms were considered targets for further analysis by reporter gene assay (miR-423-5p, miR-486-3p). In addition, we also carried out an unbiased search for all candidate miRs that could target HCN4 to find that it was a consistently predicted target for miR-27a-3p and hence this was included in the screen along with miR-1 that has been previously linked to HCN4.[1]

Prediction algorithm	Predicted HCN4-targeting miRs
Targetscan Mouse v7[11]	miR-423-5p, miR-27a-3p, miR-486-3p
PITA[12]	miR-27a-3p, miR-486-3p, miR-1a 3p/5p, Let-7e
RNA22[13]	miR-423-5p, miR-27a-3p, Let-7d

MatInspector (Genomatix, Release 8.4) was used to analyse potential transcription factor binding sites within 2 kb of the 5′ flanking region upstream of the transcription start site of the host gene of

miR-423-5p, NSRP1. On the basis of evolutionary conservation between mice and humans and scores for similarity to canonical binding sites, 79 top predicted transcription factors were selected for further expression profiling along with nine other cardiac transcription factors either known to be involved in function and/or development of the heart (transcripts studied are listed in Table S4).

Plasmids

(i) pmiRGLO-HCN4 3′-UTR. Primer pairs (forward, 5′-GCTAGCCGCTCCAAACTGCCGTCTAAT-3′; reverse, 5′-GTCGACCTCCCTCCCTCCCTCCCTCTC-3′) were used to PCR amplify 192 nt full length mouse HCN4 3′-UTR (NCBI Reference Sequence: NM_001081192.1) from mouse genomic DNA (wild type C57Bl/6). NheI and SalI sites were incorporated in the primers to facilitate cloning. 1 µl of cDNA was PCR amplified using the PfuTurbo Hotstart DNA polymerase (Agilent Technologies) in a 25 µl reaction with 2.5 µl buffer, 0.5 µl dNTPs, 1 µl of each primer (forward/reverse, 100 µM), 0.5 µl polymerase, 20 µl H_20. Cycling conditions (for 30 cycles) were 95°C for 15 min (initial denaturation), 94°C for 15 s (denaturation), 52°C for 30 s (annealing), 72°C for 45 s (extension) and 72°C for 7 min (final extension). Purification of PCR products and plasmid DNA as well as separation of DNA after restriction digests were performed by cutting bands of appropriate size from a 1.5 % agarose gel under UV-light and subsequent purification of DNA from agarose gels with Qiaquick gel extraction kit (Qiagen) according to the manufacturer's instructions. In subsequent cloning steps, 2 µg of pmiRGLO dual luciferase miR target expression vector (Promega, E1330) DNA was digested with 1 µl NheI and 1ul SalI. 8 µl of purified PCR product (amplified HCN4 3′-UTR) was also digested with 1 µl NheI and 1 µl SalI. For subsequent cloning, linearised vector DNA was dephosphorylated following which linearised plasmid DNA and inserts were purified on a 1.5% agarose gel. For ligation, 100 ng of digested vector DNA was incubated with inserts in four different molar ratios (vector to insert 1:3, 1:5, 1:7 or 1:9) along with 1 µl of 10x ligase buffer and 1 µl T4 DNA ligase overnight at 16°C. Competent *E. coli* cells (DH5α, Sigma-Aldrich) were transformed with ligated plasmids. 2 ml LB medium supplemented with ampicillin (final concentration of 100 µg/ml) was inoculated with a single bacterial colony and incubated overnight at 37°C with shaking (250 rpm). Plasmid DNA was purified using the Purelink Plasmid Kit (Thermo Fisher) according to manufacturer's instructions. For analytical restriction digest, plasmid DNA was incubated with the restriction enzymes NheI and SalI for 1.5 h at 37°C to confirm the presence of the correct ligation of 3'UTR inserts in the pmirGLO vector. Mutant HCN4 3'UTR with nucleotide substitutions for two predicted miR-423-5p binding sites was generated by GenScript (USA).

(ii) pcDNA-NKX2.5. pEntr-Nkx2-5flbio containing mouse Nkx2.5 cDNA was a gift from William Pu (Addgene plasmid # 32969). The Nkx2.5 fragment was then transferred to the vector pcDNA6.2 cLumio-DEST (Invitrogen) by using the Gateway vector system (Invitrogen) to produce pcDNA6.2-Nkx2.5 using a protocol recommended by the manufacturer.

(iii) pGL3-NSRP1. A 2.1 kb fragment upstream of the NSRP1 transcription start site, corresponding to the promoter region and encompassing predicted Nkx2.5 binding sites (given in Figure S3) was synthesised by Dundee Cell Products. The fragment was then directionally subcloned into a luciferase containing plasmid, pGL3-basic (Promega) reporter using Kpn I and Hind III cloning sites, which were incorporated to the NSRP1 promoter construct, to generate a NRSP1 promoter luciferase construct.

Cell culture, transfection and reporter assays

H9c2 cells were maintained in Dulbecco's modified Eagle's medium (DMEM) (Invitrogen) supplemented with 10% foetal bovine serum and 1% penicillin/streptomycin. For Nkx2.5 overexpression, 5 x10^5 cells /well were plated in 6-well plates 24 h prior to transfection with 3 µg pcDNA3.1-Nkx2.5 or pcDNA3.1 empty vector. For reporter assays investigating miRs, cells were seeded at a density of 10^5cells/well in 24-well plates 24 h prior to transfection and co-transfected with 500 ng HCN4 3′-UTR plasmid or mutant and 0.5-1.5 µg precursor miR or negative control plasmid. For reporter assays testing NRSP1 promoter activity, the same procedures were followed to co-transfect H9C2 cells with 1 µg of promoter-luciferase fusion plasmid pGL3 and pcDNA-Nkx2.5 or negative control. Lipofectamine 2000 (Invitrogen) was used for all transfections according to the manufacturer's instructions. Transfected cells were incubated with DNA-Lipofectamine complexes for 24 h before lysing in passive lysis buffer (Promega) for luciferase assay or washed with phosphate buffered saline (PBS) and incubated for a further 24 h with 2 ml of fresh DMEM before lysis in Trizol (Invitrogen) for RNA extraction. Luciferase activity was determined using a Luciferase Assay System (Promega) using 10 µl of cell lysate on a

luminometer (Berthold Technologies Lumat LB 9507). For each luminescence reading the injector was programmed to dispense 50 µl assay reagent after which there was a 2 s pre-measurement delay followed by a 7 s measurement period. Luciferase assays were performed in quadruplicate and repeated three times with an independent batch of cells. For miRs Firely luciferase and renilla luciferase activity were measured and data were analysed based on ratio of Firely/Renilla activity.

Western blot

HCN4 protein levels were determined by western blot using previously described methods.[14] Briefly, protein lysate was obtained by homogenising snap frozen sinus node biopsies using an MP FastPrep-24 5^{G} and 2 ml tubes containing FastPrep metal bead lysing matrix (1.4 mm) in RIPA buffer (Sigma Aldich)**.** Total protein concentration was estimated using Bradford protein assay against standard curve of bovine serum albumin (BSA; 0-0.5 mg/ml) following which samples were denatured by adding final volume of 25% SDS-sample buffer - 100 mM Tris-HCl, pH 6.8, 25% (v/v) glycerol, 10% (v/v) SDS, 10% (v/v) β-mercaptoethanol, 0.1% (w/v) bromophenol blue - and heating to 80˚C for 10 min. Samples were loaded onto a 12% stain-free SDS-polyacrylamide gel (Bio-Rad) with PreSciccion Plus (Bio-Rad) protein standards and run at 50 mV for ~50 min in SDS running buffer (25 mM Tris, 192 mM glycine, 0.1% SDS). Stain-free gels were imaged using ChemiDoc MP and then transferred to PVDF (polyvinyl difluoride) membranes using a Trans-Blot Turbo transfer system (Bio-Rad) at 15 V/0.3 mA for 15 min. PVDF membranes (activated in 100% ethanol before use) and thick filter paper were pre-wet in transfer buffer - 1x Trans-Blot Turbo transfer buffer (Bio-Rad) and 20% (v/v) ethanol. Successful transfer was confirmed by using the ChemiDoc MP. PVDF membranes were washed in TBS for 5 min and then blocked in milk-TBS-Tween (5% w/v non-fat dried Marvel milk, 0.1% v/v TBS and Tween 20) for 1 h at room temperature with gentle rocking. The membranes were then probed with the following primary antibodies for 1 h at room temperature with gentle rocking: rabbit polyclonal anti-HCN4 (Alomone labs), 1:100; rabbit polyclonal anti-actin (Sigma Aldrich), 1:1000. Following three 10 min washes in TBS-Tween, membranes were then probed with horseradish peroxidase (HRP)-linked secondary antibody (HRP-linked anti-rabbit IgG, Cell Signalling) for a further 1 h at room temperature with gentle rocking. Membranes were then washed three times for 5 min in TBS-Tween to remove unbound secondary antibody. Chemiluminescence was achieved by the addition of Clarity Western ECL substrate (Bio-Rad) in a 1:1 ratio for 5 min in the dark. Membranes were then imaged with the ChemiDoc MP. Sedentary, trained and trained+antimiR samples were run on the same gel to ensure identical exposure conditions. The chemiluminescent signal intensity was normalised to the relative quantification of the corresponding intensity of actin. Data from each replicate were normalised and averaged across replicates.

Proteomics

Mass spectrometry based proteomics experiments were performed to evaluate protein expression of selected targets in isolated sinus node biopsies from sedentary male C57BL/6J mice (n=30 pooled into 3 samples with 10 biopsies in each sample). Briefly, sinus node biopsies were collected and immediately snap frozen in liquid N_2 and stored at -80°C until processing. Cardiac proteins were extracted from the biopsies and 1 mg protein from each sample was digested as described previously.[15, 16] Peptides were desalted and fractionated by micro-flow reverse-phase ultra high pressure liquid chromatography into 12 fractions. Fractionated peptide samples were analyzed by online reversed-phase liquid chromatography coupled to a Q-Exactive Plus quadrupole Orbitrap tandem mass spectrometer. Peptide samples were separated on 15 cm fused-silica emitter columns using a 1 h multi-step linear gradient. Raw mass spectrometry data was processed using MaxQuant software (version 1.5.3.30) and proteins were identified with the built-in Andromeda search engine using a database containing all reviewed mouse SwissProt protein entries. Analysis of protein abundance was based on summed mass spectrometry-based protein intensities as determined by MaxQuant.

TUNEL assay

The right atrial wall including the sinus node of sedentary and trained mice were quickly dissected, flash frozen with liquid N_2, and stored at -80°C until processing. The tissues were cryosectioned at 12 µm thick and mounted every five sections on an adhesion glass slide. Terminal deoxynucleotidyl transferase biotin-dUTP nick-end labelling (TUNEL; Roche, 11 684 795 910) was then performed combined with standard immunohistochemistry to label the sinus node using anti-HCN4 antibody. Briefly, frozen sections were fixed in 10% neutral buffered formalin, permeabilised in 0.1% triton X-100 in PBS, and blocked with 1% BSA, followed by incubation in rabbit polyclonal

anti-HCN4 antibody (Alomone, APC-052, 1:200 dilution) at 4°C overnight. TUNEL was then carried out at 37°C for 60 min, according to the manufacturer's instruction. Cy3-conjugated anti-donkey and rabbit IgG secondary antibody (Merck Millopore, AP182C, 1:200 dilution) was added in the TUNEL reaction mix. As a positive control, sinus node sections were treated with micrococcal nuclease (ThermoFisher Scientific, EN0181, 30 U/ml). Images of HCN4-positive areas indicating the sinus node region were acquired using a laser scanning microscope (Zeiss LSM 5 PASCAL) equipped with a x40/1.0 PL Apo objective. The confocal settings were as follows: confocal aperture, 200 µm; scan speed, 1.60 µs pixel time, unidirectional; image size, 512 x 512 pixels. Images were acquired using the following conditions: 488 nm excitation and 505-530 nm emission for TUNEL, and 543 nm excitation and >560 nm emission for Cy3, respectively. To count the number of TUNEL-positive cells, images of HCN4-positive regions were combined and the contrast was enhanced using Adobe Photoshop. TUNEL positive cells per section were counted using Cell Counter of ImageJ at five different levels of the sinus node in five sedentary and trained mice.

Tissue electrophysiology

The beating rate of the isolated sinus node was determined by extracellular potential recording as described by Yamamoto *et al.*[17] In brief, animals were weighed and then killed by cervical dislocation following which a right atrial preparation encompassing the sinus node was rapidly dissected in Tyrode solution containing (in mM): 100 NaCl, 4 KCl, 1.2 $MgSO_4$, 1.2 KH_2PO_4, 1.8 $CaCl_2$, 25 $NaHCO_3$ and 10 glucose bubbled with 95% O_2 and 5% CO_2 to give a pH of 7.4. The preparation was superfused with 37°C Tyrode solution at a flow rate of 10 ml/min and extracellular potentials recorded using bipolar electrodes 100 µm in diameter. Recording electrodes interfaced with a Neurolog system (Digitimer) with low-pass and high-pass filters adjusted to optimise the signal-to-noise ratio. Extracellular potentials were continuously recorded for 20 min using a PC with a PowerLab and LabChart v7 software (ADInstruments) following which the effect of 2 mM CsCl (Sigma-Aldrich) on the beating rate was studied. The superfusing solution was changed to Tyrode solution containing 2 mM CsCl. After 15 min of treatment, the rate was recorded for 5 min. The preparation was then washed of CsCl for 20 min, during which the beating rate approached baseline values. The calculated rate was averaged over 500 beats.

Intracellular action potential recording

Intracellular action potentials were recorded in right atrial preparations containing the intact sinus node. Tissue was pinned to a specially designed chamber that allowed epicardial and endocardial contact with superfusing Tyrode solution (containing in mM: NaCl 120.3, KCl 4.0, $CaCl_2$ 1.2, $MgSO_4$ 1.3, NaH_2PO_4 1.2, $NaHCO_3$ 25.2 and glucose 11) bubbled with 95% O2 and 5% CO2 to give a pH of 7.4. Tyrode solution was circulated at 20 ml/min and tissues maintained at 37°C. The leading pacemaker site in the sinus node was mapped with bipolar extracellular electrodes as described in the preceding section. Using 3 M KCl filled sharp microelectrodes of 20-40 MΩ electrical resistance, intracellular action potentials were recorded at the leading pacemaker site of the sinus node and pectinate muscle (atrial tissue). Data acquired at 20 kHz was passed through a 10 kHz low‐pass Bessel filter and amplified 10x by Axon Instruments GeneClamp 500 amplifier (Molecular Devices Inc), digitized with Axon Instruments Digidata 1440A (Molecular Devices Inc), and recorded onto a computer using the WinEDR v3.3.6 program (Dr. J. Dempster, University of Strathclyde, Glasgow, UK). Series of five consecutive action potentials were exported to LabChart v8 software (ADInstruments) and the following action potential parameters were measured: cycle length (interval between consecutive action potential peaks, ms), maximum diastolic potential (MDP, mV), maximum upstroke velocity (dV/dt_{max}, mV/s), action potential height/amplitude (mV), action potential width (interval between consecutive maximum diastolic potentials, ms) and action potential duration (APD, ms) at 10, 50, 70 and 90% repolarization. Heart rate (beats per minute, bpm) was calculated from cycle length measurements for individual observations. GraphPad Prism 6 (GraphPad Software, Inc.) was used for statistical analysis.

Sinus node cell isolation and patch-clamp electrophysiology

Mice were killed by cervical dislocation. After quick removal of the heart, the sinus node tissue was dissected out and strips of nodal tissue were dissociated into single cells by a standard enzymatic and mechanical procedure.[18] The enzyme solution contained collagenase IV (224 U ml^{-1}, Worthington), elastase (1.42 U ml^{-1}, Sigma-Aldrich) and protease (0.45 U ml^{-1}, Sigma-Aldrich).[18] Isolated sinus node cells were stored at 4°C for the day of the experiment. I_f was recorded using a patch electrode in whole-cell mode during superfusion of a Tyrode solution containing (in mM): 140 NaCl, 5.4 KCl, 1.8 $CaCl_2$, 1 $MgCl_2$, 5 HEPES-NaOH, 10 D-glucose, pH 7.4. $BaCl_2$ (1 mM) and

$MnCl_2$ (2 mM) were added to avoid contamination from other ionic currents. The bath temperature was 35±0.5°C. The pipette solution contained (in mM): 130 K-aspartate, 10 NaCl, 2 $CaCl_2$ (pCa=7), 2 $MgCl_2$, 10 HEPES, 5 EGTA, 2 ATP(Na_2), 0.1 GTP, 5 creatine phosphate, pH 7.2. To obtain current densities, currents were measured during steps to the range −35 to −125 mV from a holding potential of −35 mV and normalised to cell capacitance. Data were acquired at 1 kHz using an Axopatch 200 amplifier and pClamp 8 (Molecular Devices, Sunnyvale, CA, USA). Data were analysed off-line using Clampfit 10 (Molecular Devices), Origin 8 (Origin Lab Corp., Northampton, MA, USA) and GraphPad Prism 6 (GraphPad Software, Inc.).

Statistical analysis

Statistical analysis was carried out using GraphPad Prism 6 or 7 (GraphPad Software, Inc.) or SPSS (IBM). Two groups were analysed using an unpaired Student's *t*-test (two tailed). When the null hypothesis of equal variance was rejected, an unpaired t-test with Welch's correction was used. If the data were not normally distributed, a non-parametric test (Mann-Whitney test) was used instead of the unpaired t-test. To compare multiple groups, an ANOVA (one- or two-way) was used in the case of normally distributed data and the Kruskal-Wallis test in the case of data not normally distributed. $P<0.05$ was regarded as significant. $0.12>P<0.05$ was regarded of potential interest and the precise P value is given. In figures, data are shown as means±SEM; asterisks indicate significance. For TLDA cards, a non-parametic Limma test was used to compare differences between sedentary and control animals.[6] Statistical analysis of the next generation sequencing data is described above.

SUPPLEMENTAL DISCUSSION

Intrinsic heart rate of untrained, young, male, human subjects

Jose and Collinson[19] reported the intrinsic heart rate for 139 male subjects (non-athletes) between the ages of 20 and 30. This is the first and largest study of the intrinsic heart rate. We have data for 10 male subjects (non-athletes) between the ages of 20 and 30. Although the intrinsic heart rate in this study (97.9±2.6 beats/min) is statistically different (Student's t test; P=0.002) from the intrinsic heart rate (105.6±0.6 beats/min) reported by Jose and Collinson,[19] it is within the mean ± 2× standard deviations from the study of Jose and Collinson.[19] The mean ± 2× standard deviations encompasses 95.4% of the data from the Jose and Collinson[19] study. Previously, we have put this forwards as a criterion for acceptance of intrinsic heart rate data.[20] Whereas the intrinsic heart rate of untrained, young, male, human subjects falls within this acceptable range in some studies, lower values (the lowest being 83.1 beats/min) have been reported in other studies;[20] in all these studies the number of human subjects was 10 or less and, in this respect, have to be considered less definitive than the study of Jose and Collinson.[19] The intrinsic heart rate of young, male subjects (non-athletes) should be approximately the same in different studies. The low intrinsic heart rates reported in some studies are likely to be the result of a technical issue, because the only factors known to decrease the intrinsic heart rate (age,[19] heart failure[21] and athletic training[e.g.22]) are unlikely to apply.

Reported evidence of exercise training-induced increase of vagal tone

Based on block of autonomic tone using atropine and propranolol, we have found no evidence of altered autonomic tone and in particular high vagal tone in human athletes (this study; Figure 1B) and exercise trained C57BL/6J mice.[1] However, again based on block of autonomic tone using atropine and propranolol, Guasch *et al.*[23] (using the Wistar rat) and Aschar-Sobbi *et al.*[24] (using the CD1 mouse) have recently reported evidence of high vagal tone following exercise training (although, strangely, Guasch *et al.*[23] stated that there was no exercise training-induced bradycardia in their study). The cause of the discrepancy is unknown, although it could be the result of an unknown technical issue, the doses of atropine and propranolol used, species used, strain of mouse used, and differences in the duration, intensity and type of training.

We have reviewed the role of high vagal tone (assessed by pharmacological block of autonomic tone) in exercise training-induced bradycardia in the human and animal models.[20] In all studies of human athletes in which the measurement of the intrinsic heart rate is deemed to be correct (see above) there is no evidence of high vagal tone.[20] In nine animal studies, the data suggests that high vagal tone accounts for 76% (mouse), 40% (rat), 43.6% (rat), 10% (rat), 0% (rat), 0% (rat), 0% (rat), 0% (rat) and 0% (rat) of the exercise training-induced bradycardia.[20] Aschar-Sobbi *et al.*[24] state that high vagal tone accounts for 100% of the exercise training-induced bradycardia in the mouse. This is a higher contribution than any of the previous studies. In

contrast, in the mouse we have argued that its contribution is 0%,[1] consistent with five of the previous studies. Also in our case, we have based our conclusion not only on autonomic blockade *in vivo* in the mouse; we have also based it on intrinsic heart rate measurements from the isolated sinus node from both the rat and mouse.[1] Furthermore, we have shown that there is an exercise training-induced downregulation of HCN4 mRNA, HCN4 protein and funny current in the rat and mouse and the exercise training-induced bradycardia is abolished on block of funny current in the mouse (this study and D'Souza *et al.*[1]).

We conclude that it is likely that downregulation of funny current is playing a role in exercise training-induced bradycardia. However, the evidence from Guasch *et al.*[23] and Aschar-Sobbi *et al.*[24] of exercise training-induced high vagal tone is difficult to refute and perhaps high vagal tone could play a role in some circumstances.

Nkx2.5 expression in the adult sinus node

While much is known about the regulatory networks at play in the embryonic development of the cardiac conduction system, the transcriptional networks maintaining function of the adult sinus node are comparatively understudied. The data presented in Online Table IV, as far as we are aware, is the first large scale transcriptomic analysis of transcription factors within the adult mouse sinus node. In Online Figure XA, data from Online Table IV are plotted to show expression levels of 88 transcription factors in the sedentary adult mouse sinus node. Transcriptionally, Nkx2.5 (red bar) is the ninth most abundant transcription factor (of the transcription factors measured). Expression levels of well-known transcription factors in the sinus node (Tbx3, Tbx18 and Shox2) are highlighted in blue for comparison. Online Figure XB shows the protein expression level of selected transcription factors (measured by mass spectrometry) in the sedentary adult mouse sinus node. This confirms the presence of Nkx2.5 in the adult mouse sinus node. Finally, in recent work, Wu *et al.*[25] found that H3K4me3 modifications (a prominent active histone mark associated with active genes) were highly enriched in the Nkx2.5 promoter in the mouse sinus node. In summary, these observations suggest that there is baseline expression of Nkx2.5 in the sinus node (which is then increased in response to exercise training).

REFERENCES

1. D'Souza A, Bucchi A, Johnsen AB, Logantha SJ, Monfredi O, Yanni J, Prehar S, Hart G, Cartwright E, Wisloff U, Dobryznski H, DiFrancesco D, Morris GM and Boyett MR. Exercise training reduces resting heart rate via downregulation of the funny channel HCN4. *Nature Communications*. 2014;5:3775.
2. Liu W, Zi M, Jin J, Prehar S, Oceandy D, Kimura TE, Lei M, Neyses L, Weston AH, Cartwright EJ and Wang X. Cardiac-specific deletion of *Mkk4* reveals its role in pathological hypertrophic remodeling but not in physiological cardiac growth. *Circulation Research*. 2009;104:905-14.
3. Dirkx E, Gladka MM, Philippen LE, Armand AS, Kinet V, Leptidis S, El Azzouzi H, Salic K, Bourajjaj M, da Silva GJ, Olieslagers S, van der Nagel R, de Weger R, Bitsch N, Kisters N, Seyen S, Morikawa Y, Chanoine C, Heymans S, Volders PG, Thum T, Dimmeler S, Cserjesi P, Eschenhagen T, da Costa Martins PA and De Windt LJ. Nfat and miR-25 cooperate to reactivate the transcription factor Hand2 in heart failure. *Nature Cell Biology*. 2013;15:1282-93.
4. Kozomara A and Griffiths-Jones S. miRBase: annotating high confidence microRNAs using deep sequencing data. *Nucleic Acids Res*. 2014;42:D68-73.
5. Bang C, Batkai S, Dangwal S, Gupta SK, Foinquinos A, Holzmann A, Just A, Remke J, Zimmer K, Zeug A, Ponimaskin E, Schmiedl A, Yin X, Mayr M, Halder R, Fischer A, Engelhardt S, Wei Y, Schober A, Fiedler J and Thum T. Cardiac fibroblast-derived microRNA passenger strand-enriched exosomes mediate cardiomyocyte hypertrophy. *Journal of Clinical Investigation*. 2014;124:2136-46.
6. Jeanmougin M, de Reynies A, Marisa L, Paccard C, Nuel G and Guedj M. Should we abandon the t-test in the analysis of gene expression microarray data: a comparison of variance modeling strategies. *PloS one*. 2010;5:e12336.
7. Bolger AM, Lohse M and Usadel B. Trimmomatic: a flexible trimmer for Illumina sequence data. *Bioinformatics*. 2014;30:2114-20.

8. Kim D, Pertea G, Trapnell C, Pimentel H, Kelley R and Salzberg SL. TopHat2: accurate alignment of transcriptomes in the presence of insertions, deletions and gene fusions. *Genome Biology*. 2013;14:R36.
9. Anders S, Pyl PT and Huber W. HTSeq - a Python framework to work with high-throughput sequencing data. *Bioinformatics*. 2015;31:166-9.
10. Anders S and Huber W. Differential expression analysis for sequence count data. *Genome Biology*. 2010;11:R106.
11. Miranda KC, Huynh T, Tay Y, Ang YS, Tam WL, Thomson AM, Lim B and Rigoutsos I. A pattern-based method for the identification of microRNA binding sites and their corresponding heteroduplexes. *Cell*. 2006;126:1203-17.
12. Kertesz M, Iovino N, Unnerstall U, Gaul U and Segal E. The role of site accessibility in microRNA target recognition. *Nat Genet*. 2007;39:1278-84.
13. Agarwal V, Bell GW, Nam JW and Bartel DP. Predicting effective microRNA target sites in mammalian mRNAs. *eLife*. 2015;4.
14. Pinali C, Bennett HJ, Davenport JB, Caldwell JL, Starborg T, Trafford AW and Kitmitto A. Three-dimensional structure of the intercalated disc reveals plicate domain and gap junction remodeling in heart failure. *Biophysics Journal*. 2015;108:498-507.
15. Lundby A, Rossin EJ, Steffensen AB, Acha MR, Newton-Cheh C, Pfeufer A, Lynch SN, Consortium QTIIG, Olesen SP, Brunak S, Ellinor PT, Jukema JW, Trompet S, Ford I, Macfarlane PW, Krijthe BP, Hofman A, Uitterlinden AG, Stricker BH, Nathoe HM, Spiering W, Daly MJ, Asselbergs FW, van der Harst P, Milan DJ, de Bakker PI, Lage K and Olsen JV. Annotation of loci from genome-wide association studies using tissue-specific quantitative interaction proteomics. *Nature Methods*. 2014;11:868-74.
16. Lundby A, Andersen MN, Steffensen AB, Horn H, Kelstrup CD, Francavilla C, Jensen LJ, Schmitt N, Thomsen MB and Olsen JV. In vivo phosphoproteomics analysis reveals the cardiac targets of beta-adrenergic receptor signaling. *Science Signaling*. 2013;6:rs11.
17. Yamamoto M, Honjo H, Niwa R and Kodama I. Low frequency extracellular potentials recorded from the sinoatrial node. *Cardiovascular Research*. 1998;39:360-372.
18. DiFrancesco D, Ferroni A, Mazzanti M and Tromba C. Properties of the hyperpolarizing-activated current (i_f) in cells isolated from the rabbit sino-atrial node. *Journal of Physiology*. 1986;377:61-88.
19. Jose AD and Collison D. The normal range and determinants of the intrinsic heart rate in man. *Cardiovascular Research*. 1970;4:160-167.
20. Boyett MR, D'Souza A, Zhang H, Morris GM, Dobrzynski H and Monfredi O. Viewpoint: Is the resting bradycardia in athletes the result of remodelling of the sinoatrial node rather than high vagal tone? *Journal of Applied Physiology*. 2013;114:1351-1355.
21. Jose AD and Taylor RR. Autonomic blockade by propranolol and atropine to study intrinsic myocardial function in man. *Journal of Clinical Investigation*. 1969;48:2019-2031.
22. Katona PG, McLean M, Dighton DH and Guz A. Sympathetic and parasympathetic cardiac control in athletes and nonathletes at rest. *Journal of Applied Physiology*. 1982;52:1652-1657.
23. Guasch E, Benito B, Qi X, Cifelli C, Naud P, Shi Y, Mighiu A, Tardif JC, Tadevosyan A, Chen Y, Gillis MA, Iwasaki YK, Dobrev D, Mont L, Heximer S and Nattel S. Atrial fibrillation promotion by endurance exercise: demonstration and mechanistic exploration in an animal model. *Journal of the American College of Cardiology*. 2013;62:68-77.
24. Aschar-Sobbi R, Izaddoustdar F, Korogyi AS, Wang Q, Farman GP, Yang F, Yang W, Dorian D, Simpson JA, Tuomi JM, Jones DL, Nanthakumar K, Cox B, Wehrens XH, Dorian P and Backx PH. Increased atrial arrhythmia susceptibility induced by intense endurance exercise in mice requires TNF$\propto$. *Nature Communications*. 2015;6:6018.
25. Wu M, Peng S, Yang J, Tu Z, Cai X, Cai CL, Wang Z and Zhao Y. Baf250a orchestrates an epigenetic pathway to repress the Nkx2.5-directed contractile cardiomyocyte program in the sinoatrial node. *Cell Research*. 2014;24:1201-13.

AUTHOR CONTRIBUTIONS

Obtained funding and design and supervision of study: Boyett, D'Souza, Dobrzynski, Morris, Cartwright, Monfredi
Human study: Morris, Pearman, Coulson, McPhee
In vivo studies: D'Souza, Cox, Johnsen, Wisloff, Da Costa Martins, Cartwright
qPCR, next generation sequencing and computational predictions: D'Souza, Elliott, Nakao
Cell culture, transfection and reporter assays: D'Souza, Oceandy, Robertson
Western blot: D'Souza, Dobrzynski, Zhang, Bennett, Kitmitto
Proteomics: Lundby, Linscheid, Poulsen, Logantha, Boyett
TUNEL assay: Nakao
Tissue electrophysiology: D'Souza
Intracellular action potential recording: Logantha
Patch clamp: WangProduction of figures and manuscript writing: D'Souza, Boyett
Proof reading: All authors

Online Table I. Characteristics of human subjects. All athletes participated in endurance sports (running, n=4; triathalon, n=3; cycling n=1). BMI, body mass index; SDNN, standard deviation of normal to normal beats; cSDNN, heart rate corrected SDNN.

	Control subjects (n = 10)	Athletes (n = 8)	P value
**Gender**	male	male	
**Age (years)**	21.0 ± 0.7	25.9 ± 1.2	**0.002**
**Weight (kg)**	69.5 ± 1.9	69.8 ± 3.8	0.95
**BMI ($kg.m^{-2}$)**	21.5 ± 0.7	22.0 ± 078	0.61
**Systolic blood pressure (mmHg)**	126.6 ± 3.8	120.9 ± 5.7	0.40
**Diastolic blood pressure (mmHg)**	70.9 ± 3.4	65.4 ± 4	0.30
**Heart rate(beats/min)**	62.5 ± 1.9	49.2 ± 2.9	**0.002**
**SDNN (ms)**	64.7 ± 7.3	72.1 ± 9.1	0.43
**cSDNN (ms)**	185.6 ± 18.6	173.6 ± 23.7	0.69

Online Table II. Intracellular action potential parameters from control mice (sinus node from 4 mice and right atrium from 3 mice) and exercise trained mice (sinus node from 8 mice and right atrium from 5 mice). APD_{10} etc., action potential duration at 10% repolarization etc. Significant differences highlighted by P values in bold font.

	Control	Trained	P value
**Sinus node (control, n=65 impalements; trained, n=43)**			
**Cycle length (ms)**	0.12±0.0006	0.12±0.0011	**0.0001**
**Spontaneous rate (beats/min)**	521±2.9	498±4.9	**0.0001**
**Maximum diastolic potential (mV)**	-53.3±1.1	-50.0±1.2	**0.046**
**Height (mV)**	44.8±1.7	39.40±1.1	**0.0087**
**APD_{10} (ms)**	9.1±0.4	9.4±0.2	0.45
**APD_{50} (ms)**	28.3±0.5	28.6±0.4	0.67
**APD_{70} (ms)**	38.1±0.6	39.3±0.6	0.18
**APD_{90} (ms)**	66.0±1.2	70.2±1.3	**0.021**
**Action potential width (ms)**	95.4±0.9	100.9±1.4	**0.0007**
**Right atrium (control, n=12 impalements; trained, n=26 impalements)**			
**Maximum diastolic potential (mV)**	-80.9±1.2	-79.5±0.8	0.32
**Height (mV)**	96.7±1.6	94.3±1.5	0.32
**APD_{10} (ms)**	1.8±0.1	1.7±0.1	0.83
**APD_{50} (ms)**	11.7±0.4	10.7±0.5	0.17
**APD_{70} (ms)**	19.7±0.5	17.3±0.8	**0.017**
**APD_{90} (ms)**	35.3±0.5	31.2±1.2	**0.0029**
**Action potential width (ms)**	96.8±3.9	98.5±3.6	0.77

Online Table III. Differentially expressed miRs in sedentary and trained mice determined by next generation sequencing. Significant FDR-corrected P values in bold font.

Mature miR	Sedentary mice (DESeq normalised read count)	Trained mice (DESeq normalised read count)	Fold change (trained/ sedentary)	P value	FDR corrected P value
mmu-miR-5099	22.21487273	489.4265862	22.03148279	0.00000	**0.00000**
mmu-miR-423-5p	313.9750531	1219.565294	3.884274506	0.00000	**0.00001**
mmu-miR-486-3p	156.2892445	607.1240921	3.88461851	0.00000	**0.00003**
mmu-let-7d-3p	1235.448761	3484.464362	2.820403786	0.00001	**0.00187**
mmu-let-7e-5p	755.0028997	1800.342372	2.384550274	0.00031	**0.03533**
mmu-miR-181b-5p	278.6639706	674.6823965	2.421132503	0.00035	**0.03533**
mmu-miR-10b-5p	421.1952422	69.86093779	0.165863549	0.00035	**0.03533**
mmu-miR-676-3p	350.2394139	879.8612085	2.512170743	0.00040	**0.03533**
mmu-miR-92a-1-5p	7.942430494	40.31434913	5.075820199	0.00072	0.05695
mmu-miR-30c-2-3p	53.09431388	144.7153584	2.725628185	0.00142	0.10178
mmu-miR-181b-5p	322.0964351	692.1797756	2.148983038	0.00198	0.12856
mmu-miR-132-3p	18.19780033	55.98987347	3.076738533	0.00237	0.13843
mmu-miR-125b-1-3p	57.21390926	149.4144141	2.61150507	0.00252	0.13843
mmu-let-7b-5p	3230.518751	6635.618825	2.054041266	0.00386	0.18699
mmu-let-7c-5p	4882.488078	9682.89278	1.983188208	0.00392	0.18699
mmu-let-7c-5p	4830.155427	9473.284502	1.961279434	0.00454	0.20266
mmu-miR-200b-3p	30.59796659	2.536806286	0.082907676	0.00572	0.23647
mmu-miR-212-5p	22.91091627	62.99404349	2.749520917	0.00624	0.23647
mmu-miR-744-5p	291.9365094	947.0853346	3.244148313	0.00628	0.23647
mmu-let-7d-5p	1811.726444	3458.665196	1.909043834	0.00669	0.23913
mmu-miR-1249-3p	37.47564173	96.24148751	2.568107791	0.00876	0.29838
mmu-let-7a-5p	4387.689636	8066.421029	1.838421059	0.01006	0.31336
mmu-miR-6240	103.1427418	232.1333021	2.250602398	0.01008	0.31336
mmu-let-7a-5p	4333.114144	7889.456427	1.820735888	0.01079	0.32134
mmu-miR-5126	2.696936877	15.75565412	5.842055203	0.01285	0.36122
mmu-let-7f-5p	2713.767599	4856.736179	1.789665475	0.01314	0.36122
mmu-let-7f-5p	2436.096432	4328.468006	1.776804871	0.01463	0.37916
mmu-miR-10a-5p	1161.619985	543.2343346	0.467652366	0.01580	0.37916
mmu-miR-483-3p	1.571038647	11.09386871	7.06148683	0.01621	0.37916
mmu-miR-1940	9.943323837	34.78604993	3.498432768	0.01630	0.37916
mmu-miR-155-5p	23.75533994	56.56196019	2.381020871	0.02125	0.47476
mmu-miR-193b-3p	110.9863584	14.5344591	0.130957167	0.02673	0.56202
mmu-miR-27a-3p	344.6710656	155.8005298	0.452026716	0.02787	0.56935
mmu-miR-223-3p	40.18786432	8.282510982	0.206094828	0.03733	0.74143
mmu-miR-1306-5p	2.978411435	15.05498902	5.054704277	0.04746	0.90606

Online Table IV. List of potential *NSRP1*-targeting transcription factors measured in the sinus node using Taqman Low density array cards. Significant differences highlighted by P values in bold font.

	Sedentary		Trained		
**mRNA**	**Mean**	**SEM**	**Mean**	**SEM**	**P value**
**Predicted by MatInspector**					
Zfp354c	1.60E-01	2.89E-02	4.20E-01	8.86E-02	**0.008446**
Helt	1.69E-04	6.57E-05	3.53E-03	2.18E-03	**0.010665**
Six3	1.69E-04	6.57E-05	6.07E-02	3.91E-02	**0.016301**
Patz1	2.51E-01	4.44E-02	4.73E-01	5.95E-02	**0.016376**
Mitf	9.03E-01	2.95E-01	1.86E+00	3.39E-01	**0.021262**
Cdx2	1.69E-04	6.57E-05	1.28E-03	8.71E-04	**0.025429**
Hsf2	2.97E-01	3.66E-02	5.87E-01	1.36E-01	**0.031703**
Pdx1	1.69E-04	6.57E-05	4.37E-04	1.57E-04	**0.034216**
Spz1	1.69E-04	6.57E-05	4.37E-04	1.57E-04	**0.034216**
Foxp1	6.54E-01	6.19E-02	1.11E+00	1.67E-01	**0.035768**
Nkx2.5	1.02E+00	3.55E-01	2.15E+00	3.73E-01	**0.041394**
Tead4	5.68E-02	9.45E-03	9.60E-02	1.04E-02	**0.041491**
Stat3	1.03E+00	1.80E-01	1.80E+00	2.84E-01	**0.042595**
Cebpe	3.34E-02	1.19E-02	4.37E-04	1.57E-04	0.057859
Atf1	2.01E-01	4.19E-02	7.91E-02	1.69E-02	0.058164
Tcf3	3.67E-03	9.86E-04	3.31E-02	2.50E-02	0.061033
Sp1	6.67E-01	9.47E-02	1.07E+00	1.43E-01	0.070278
Hoxa9	6.95E-03	2.10E-03	2.97E-03	7.65E-04	0.074478
Pitx2	9.98E-02	1.13E-02	1.58E-01	2.51E-02	0.093084
Arid5a	8.09E-02	7.66E-03	1.27E-01	2.14E-02	0.103987
Sall2	2.16E-04	7.01E-05	4.37E-04	1.57E-04	0.104569
Klf15	1.39E+00	2.82E-01	3.36E+00	1.12E+00	0.114708
Nanog	4.90E-03	2.87E-03	1.70E-03	1.20E-03	0.123074
Zfp217	7.52E-02	1.53E-02	1.21E-01	2.19E-02	0.142784
Gabpa	3.06E+00	3.75E-01	5.13E+00	1.27E+00	0.149457
Hic1	4.06E-03	2.24E-03	1.32E-01	1.28E-01	0.158815
Tfap2a	2.13E-02	3.54E-03	3.78E-02	1.06E-02	0.162104
Lef1	4.28E-03	7.25E-04	1.08E-02	3.32E-03	0.162278
Klf7	4.21E-01	2.78E-01	1.07E-01	2.15E-02	0.166922
Esrra	5.99E-01	6.75E-02	8.59E-01	1.37E-01	0.185812
Nfatc2	1.41E-01	3.01E-02	2.49E-01	8.32E-02	0.216844
Arid1a	8.23E-02	1.68E-02	5.63E-02	5.22E-03	0.233172
Zfp384	1.29E-01	2.37E-02	1.03E-01	2.55E-02	0.241921
Yy1	5.11E-01	4.11E-02	7.03E-01	1.18E-01	0.302407
Atf6	5.64E-01	5.84E-02	5.72E-01	1.53E-01	0.306556
Gata1	7.68E-03	2.41E-03	6.18E-03	2.94E-03	0.323467
Csrnp3	1.51E-02	5.26E-03	9.08E-03	1.86E-03	0.331801
Grhl2	2.61E-02	2.37E-02	6.46E-02	4.41E-02	0.349373
Pou2f1	8.36E-01	1.77E-01	1.28E+00	3.66E-01	0.362792
Runx3	2.83E-02	7.21E-03	2.52E-02	1.41E-02	0.407731
Satb1	4.42E-01	7.15E-02	5.67E-01	1.04E-01	0.421099

Online Table IV (continued).

	Sedentary		Trained		
**mRNA**	**Mean**	**SEM**	**Mean**	**SEM**	**P value**
**Predicted by MatInspector**					
Pou4f3	6.39E-02	6.38E-02	1.52E-02	1.47E-02	0.422668
Pou1f1	1.23E-02	1.19E-02	4.67E-02	4.63E-02	0.446538
Zfx	2.05E-02	4.25E-03	2.44E-02	6.31E-03	0.466673
Irx3	7.73E-02	4.40E-02	3.64E-02	1.79E-02	0.478955
LIZfp239	7.25E-02	1.13E-02	7.32E-02	1.46E-02	0.503171
Sox6	2.04E-01	1.34E-02	2.25E-01	6.12E-02	0.511482
Mef2c	6.16E-01	7.84E-02	7.85E-01	1.73E-01	0.513938
Isl2	8.31E-02	6.57E-02	7.60E-02	6.64E-02	0.524187
Gfi1	1.09E-02	7.17E-03	1.34E-02	1.30E-02	0.567992
E2f1	2.31E-02	3.63E-03	2.46E-02	6.81E-03	0.580365
Dmtf1	1.30E-01	2.59E-02	1.87E-01	6.50E-02	0.584297
Hnf4a	1.89E-04	7.24E-05	9.13E-04	4.79E-04	0.613491
Insm2	8.87E-03	5.69E-03	5.02E-03	1.88E-03	0.614452
Hmbox1	1.13E-01	2.12E-02	1.22E-01	2.29E-02	0.619181
Zic3	1.82E-02	1.80E-02	1.54E-03	1.09E-03	0.622457
Rfx5	1.45E+00	3.21E-01	5.18E+00	1.54E+00	0.645909
Pax4	4.94E-02	3.88E-02	4.27E-02	3.84E-02	0.666232
Hltf	3.42E-01	3.49E-02	3.79E-01	5.85E-02	0.717274
Irf3	2.71E-01	1.00E-01	2.36E-01	3.23E-02	0.728975
Six2	1.53E-02	5.42E-03	1.46E-02	3.05E-03	0.732889
Plag1	5.90E-02	9.81E-03	5.97E-02	2.17E-02	0.739622
Zscan21	1.01E-01	2.23E-02	2.22E-01	5.05E-02	0.747869
Sall1	9.02E-03	2.60E-03	6.42E-03	1.73E-03	0.760965
Zbtb14	4.58E-02	8.27E-03	5.04E-02	1.18E-02	0.771122
Pax6	1.30E-01	1.29E-01	4.37E-04	1.57E-04	0.772495
Gli3	5.13E-02	1.11E-02	5.93E-02	1.35E-02	0.772616
Klf4	1.84E+00	4.39E-01	1.40E+00	4.12E-01	0.778102
Nr5a2	4.22E-03	1.57E-03	4.22E-03	1.96E-03	0.778849
Mzf1	7.69E-03	2.79E-03	6.75E-03	1.25E-03	0.811114
Nfkb2	2.56E-01	7.79E-02	2.67E-01	8.14E-02	0.857029
Foxh1	3.41E-02	2.78E-02	7.94E-03	5.42E-03	0.858473
Ikzf5	3.30E-01	6.57E-02	3.22E-01	3.27E-02	0.86262
Prrx2	1.81E-02	2.51E-03	3.21E-02	1.17E-02	0.863999
Smad3	2.20E-01	7.46E-02	2.23E-01	6.69E-02	0.870408
Rbpj	3.49E-01	1.26E-01	2.82E-01	4.66E-02	0.950006
Nfe2l1	8.02E-01	1.40E-01	7.90E-01	2.03E-01	0.950954
Zfp410	3.67E-01	7.91E-02	3.85E-01	8.60E-02	0.956282
Myt1l	3.75E-03	1.43E-03	3.97E-03	2.66E-03	0.992775

Online Table IV (continued).

	Sedentary		Trained		
**mRNA**	**Mean**	**SEM**	**Mean**	**SEM**	**P value**
**Predicted by MatInspector**					
**Known regulators of cardiac function/development**					
Tbx5	4.02E+00	9.96E-01	9.43E+00	1.73E+00	**0.013147**
Rest	2.25E-01	4.29E-02	5.24E-01	1.43E-01	**0.040982**
Hand2	1.92E+00	6.53E-01	3.61E+00	7.98E-01	0.076046
Tbx18	4.11E-01	1.06E-01	5.65E-01	1.05E-01	0.204897
Gata4	1.43E+00	2.54E-01	1.79E+00	2.09E-01	0.299239
Isl1	1.91E-01	5.82E-02	1.14E-01	2.35E-02	0.304207
Shox2	7.02E-02	2.42E-02	7.02E-02	1.20E-02	0.451147
Stat5a	3.04E-01	9.58E-02	3.27E-01	4.78E-02	0.454078
Tbx3	4.43E-01	1.75E-01	3.00E-01	5.70E-02	0.955335

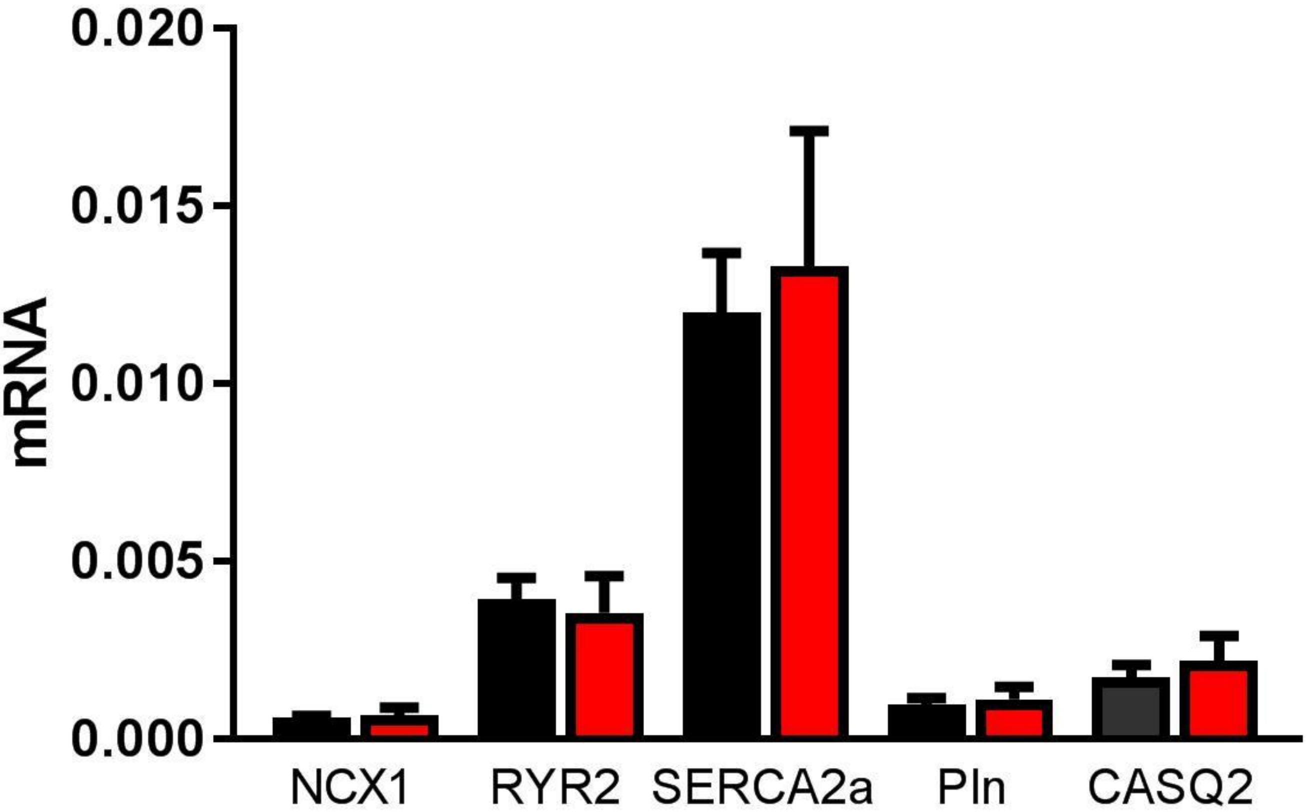

Online Figure I. mRNA expression levels for key proteins involved in the Ca^{2+} clock pacemaker mechanism in sinus node of sedentary (black bars) and trained (red bars) mice (n=5/5).

```
157    CTGCCCTCGCCCTCT--CCCTCG   CDR
            ||||| ||||  |||||:
       TTTCAGAGCGAGAGACGGGGAGT
1036   TTCATCTC-CTCCATCCCTGTCG   CDR
           |||| |||  | ||: ||:
       TTTCAGAGCGAGAGACGGGGAGT
1602   GGAG-C-AGTAC-ATGTCCTTCC   CDR
       ::|| |  |: |  ||:||:||
       TTTCAGAGCGAGAGACGGGGAGT
2519   CTTCACCCGCCAGCAGCCCCTCA   CDR
              |||   | ||||||||
       TTTCAGAGCGAGAGACGGGGAGT
2584   CTGG-CTGGATTCTCTGCACCTCC  CDR
         :| || |  :||||||| ||||
       TTTCAGAGC-GAGAGACGGGGAGT
3307   AGGGCCTCGC-CTC-ACTCCTCA   CDR
       |::| ||||| |||  |:|||||
       TTTCAGAGCGAGAGACGGGGAGT
3604   TGAG-CTGGGCCCTCCCTCCCTCT  3′ -UTR
        :|| || | | |||  :|||||
       TTTCAGAGC-GAGAGACGGGGAGT
3633   TCTTGCTC-CTTTCT-TCCTTCA   3′ -UTR
            ||| ||:||| :||:|||
       TTTCAGAGCGAGAGACGGGGAGT
```

Online Figure II. miR-423-5p binding sites on HCN4 exons (CDR, coding regions) and 3′-UTR predicted by RNA22.

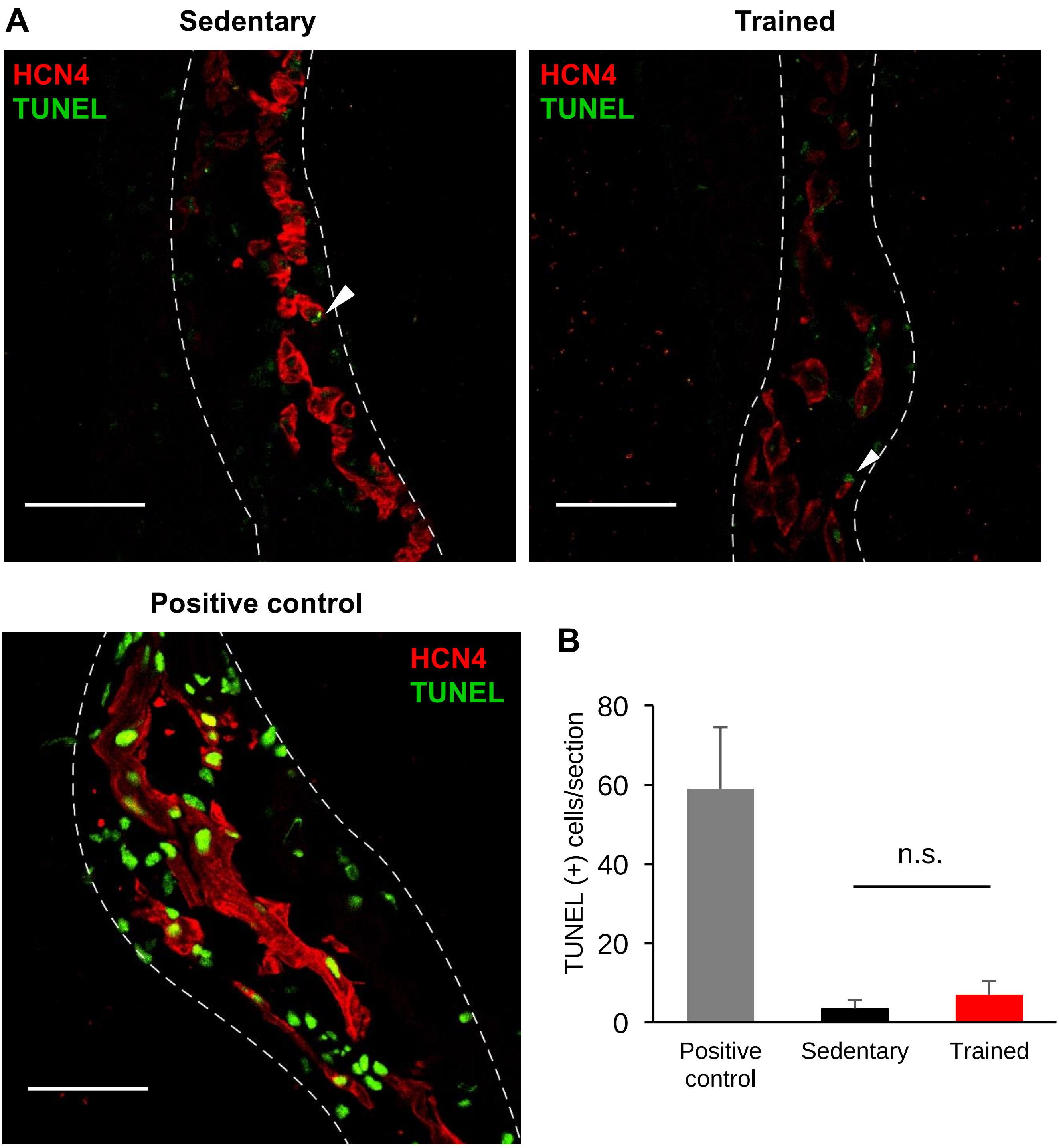

Online Figure III. TUNEL-staining of apoptotic cells in the sinus node of sedentary and trained mice. A, Sections through the sinus node treated with TUNEL (green signal; marker for apoptotic cells) and immunolabelled for HCN4 (red signal; marker for sinus node cells). Sections through the sinus node of sedentary and trained mice shown. A section though the sinus node treated with micrococcal nuclease (triggers apoptosis) also shown as a positive control. Arrowheads indicate TUNEL-positive cells. Scale bars=50 μm. **B**, Average number of TUNEL-positive cells in micrococcal nuclease-treated sinus node sections (positive control) and sinus node sections from sedentary and trained mice. (n=5/5) There were only a few apoptotic cells in the sinus node of sedentary and trained mice (3.48±2.16 versus 6.92±3.48 cells/section; P=0.4297) in contrast to the micrococcal nuclease-treated sinus node sections (59.0±15.5; P=0.001). n.s., not significant.

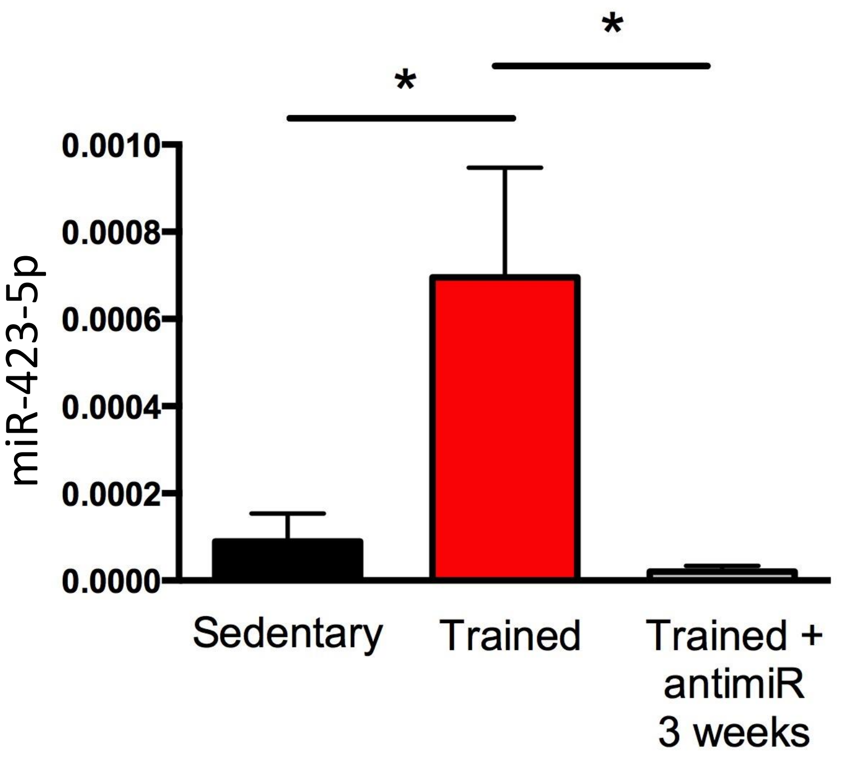

Online Figure IV. Effect of antimiR in trained animals after three weeks. miR-423-5p mRNA (normalised to RNU1A1) determined by qPCR in vehicle-injected sedentary mice, vehicle-injected trained mice and antimiR-injected trained mice three weeks after the last injection of the vehicle or antimiR (*n*=6/5/5). During this additional 3-week period, training of the mice continued. *significantly different (P<0.05).

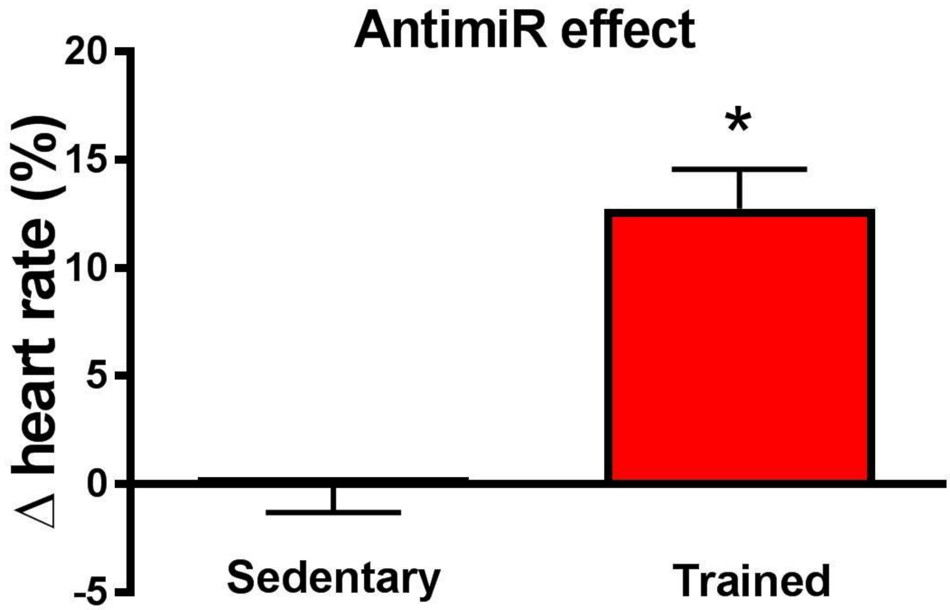

Online Figure V. Effect of antimiR on the heart rate in conscious sedentary and conscious trained mice (n=5/12). The change in heart rate caused by the antimiR is shown (calculated as the difference in heart rate, measured in the conscious mouse, before and 24 h after the third antimiR injection (day 28 of swim training). *significantly different from sedentary data (P<0.05).

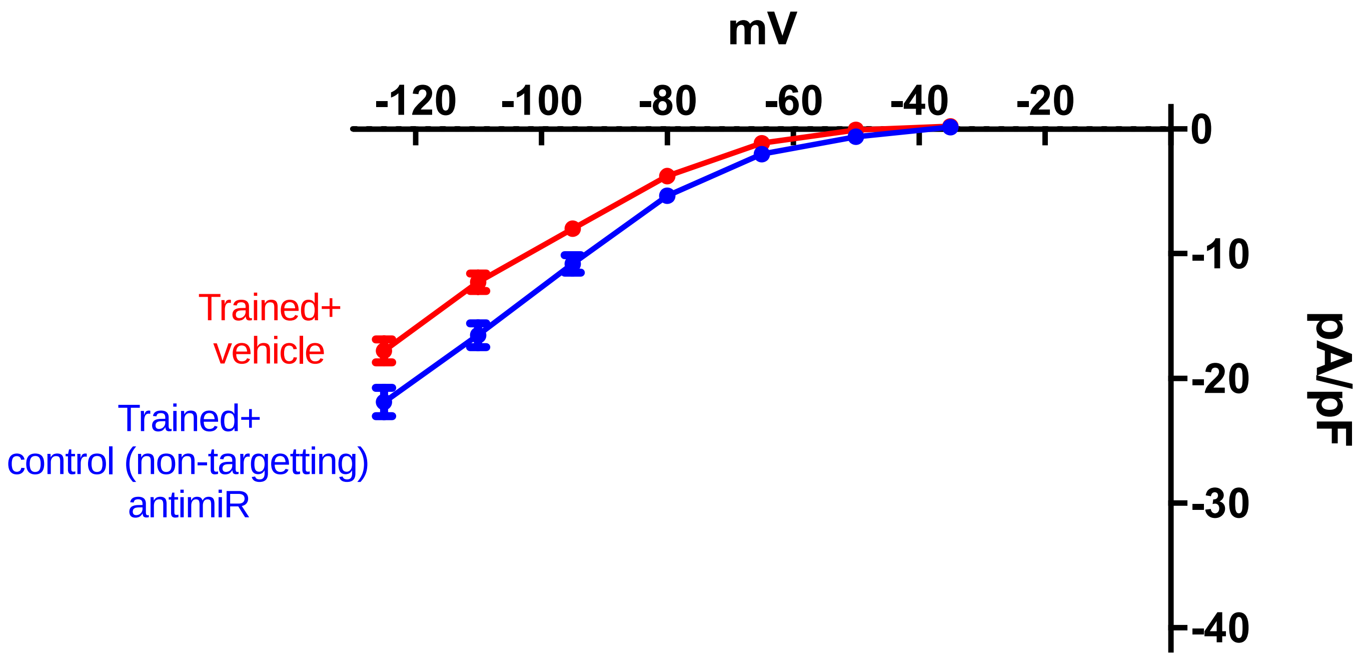

Online Figure VI. A control (non-targeting) antimiR has little effect on I_f in trained mice. Mean current-voltage relationships for I_f from vehicle-treated trained (n=47 cells/5 animals) and control (non-targeting) antimiR-treated trained (n=37 cells/4 animals) mice.

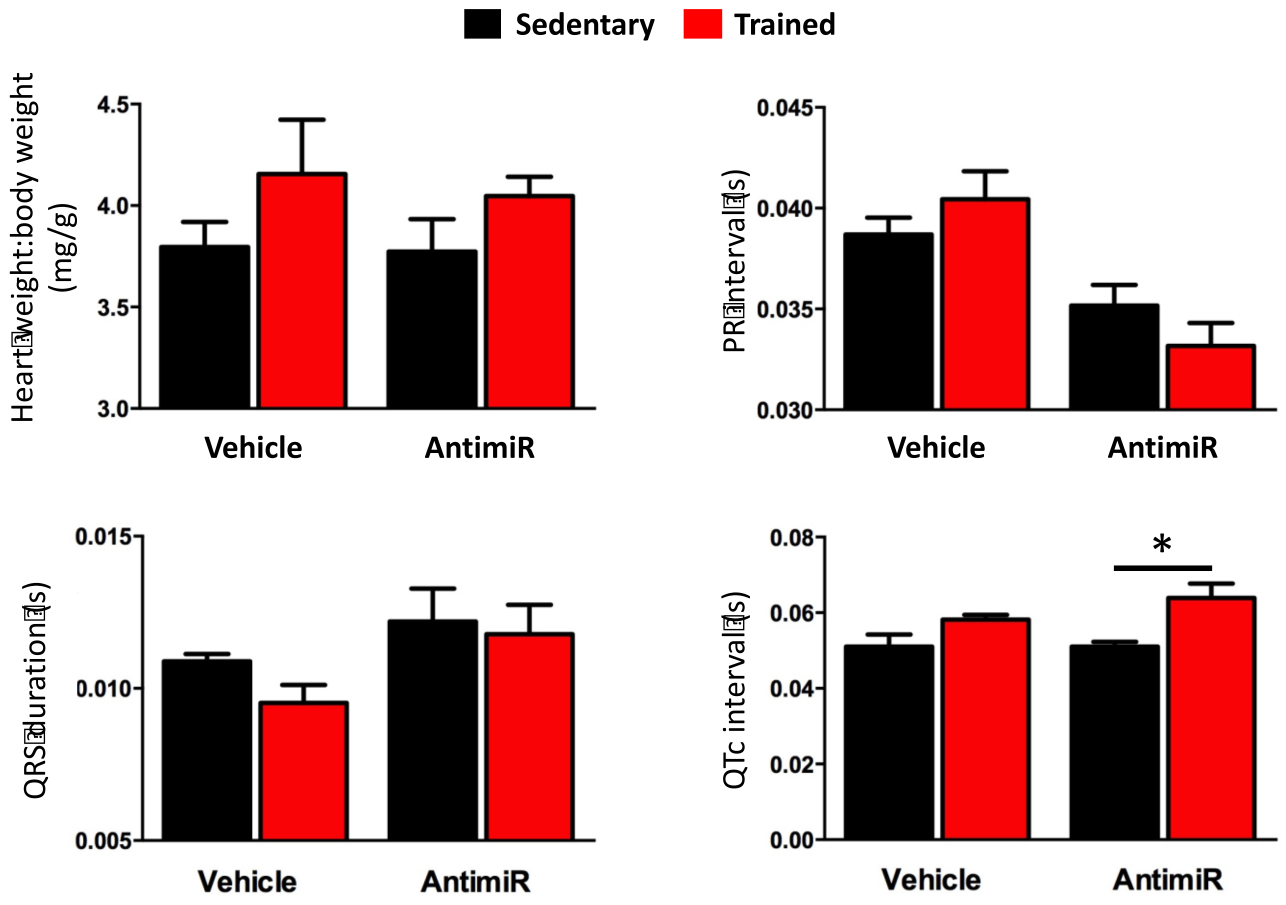

Online Figure VII. The antimiR has little or no effect on heart weight:body weight ratio, PR interval, QRS duration and QTc interval of sedentary or trained mice. n=5/5/5/6 for heart weight:body weight ratio. ECG parameters measured under isofluorane anaesthesia. Continuous 100 beat recordings were analysed and averaged. n=10/10/5/11 for ECG parameters. *significantly different (P<0.05).

	Mouse NSRP1; Chromosome 11
Nkx2.5 binding sites	77080375- ATAAATAATTGTTTAAAAA (1/0.88)
	77079441- GTGTCTGAGTGGTTTGTAC (1/0.99)

Online Figure VIII. Nkx2.5 binding sites in 2 kb 5' flanking region of the miR-423 host gene NSRP1 predicted by MatInspector. Numbers before sequences represent the position of the binding sites in the genome. Numbers in brackets indicate similarity scores for predicted Nkx2.5 binding motifs to canonical core binding sites and overall matrix for Nkx2.5 action respectively.

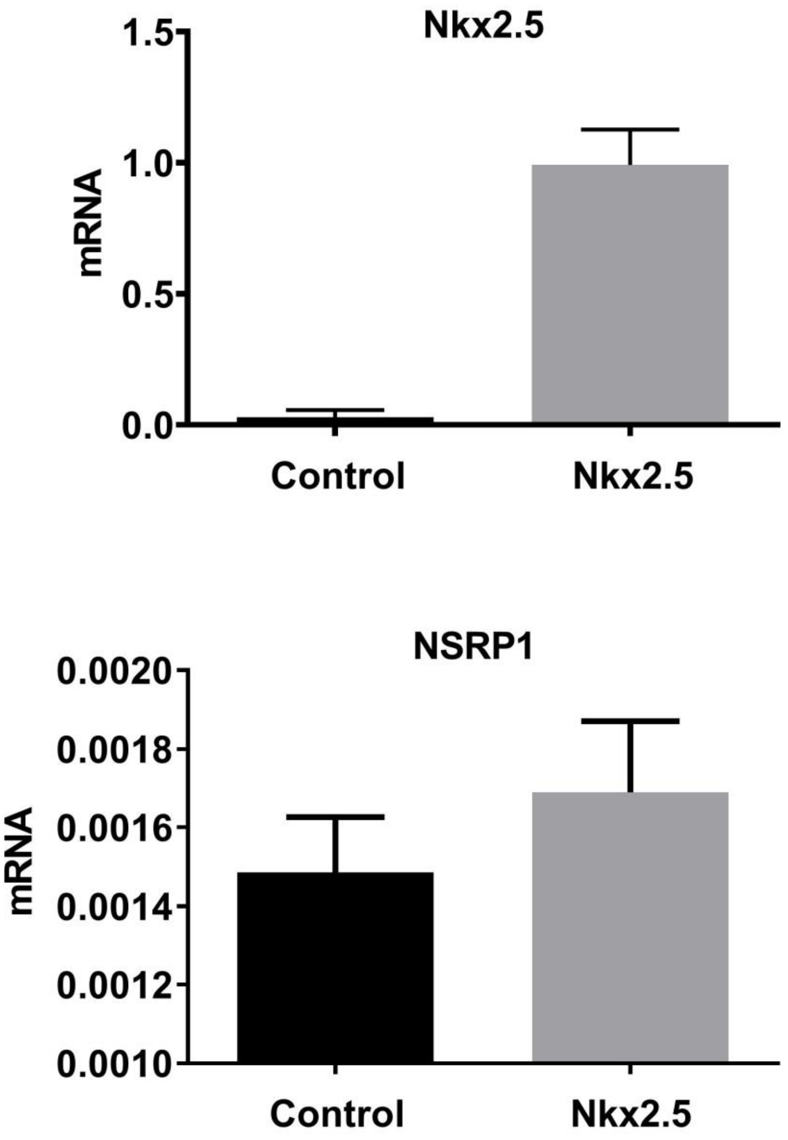

Online Figure IX. Effect of overexpression of Nkx2.5 on mRNA levels of Nkx2.5 and NSRP1 in H9c2 cells. Expression is shown in vehicle-transfected cells (control) or cells transfected with Nkx2.5.

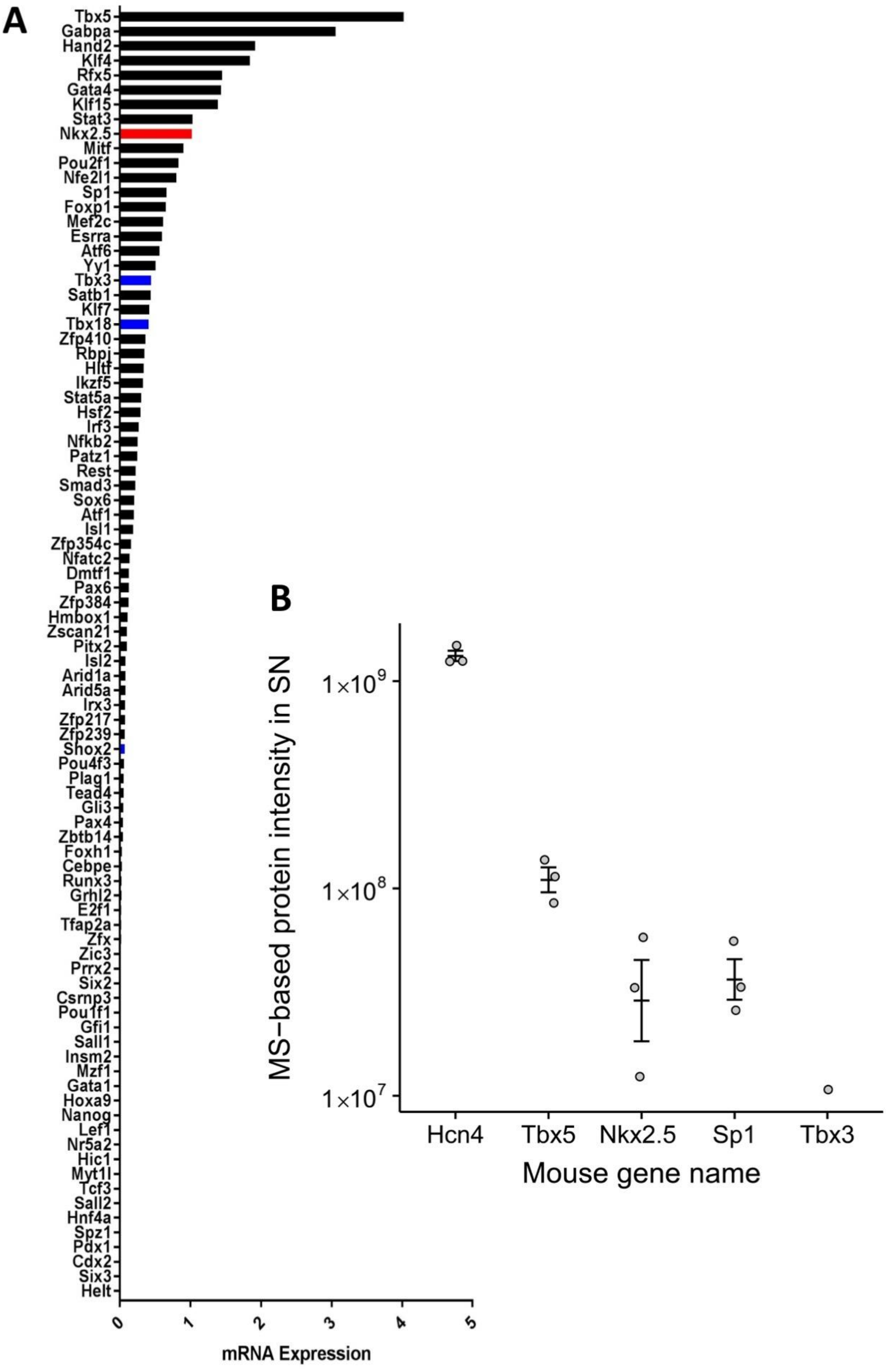

Online Figure X. Expression of Nkx2.5 in the sedentary adult mouse sinus node. A, mRNA expression level of 88 transcription factors in the sedentary adult mouse sinus node (n=8) measured using Taqman low density array cards and normalised to expression level of housekeeping genes GAPDH and TBP. Nkx2.5 is highlighted in red and, for comparison, Tbx3, Tbx18 and Shox2 are highlighted in blue. **B**, Protein expression level (on logarithmic scale) of selected transcription factors, including Nkx2.5, in the sedentary adult mouse sinus node (n=3 cohorts of 10 mice) measured using high-resolution mass spectrometry. Protein expression level of HCN4 also shown. Indivdual data points (for the three cohorts of mice) as well as means±SEM shown. MS, mass spectrometry. SN, sinus node.
